# A fractional-order model for optimizing combination therapy in heterogeneous lung cancer: integrating immunotherapy and targeted therapy to minimize side effects

**DOI:** 10.1038/s41598-024-66531-x

**Published:** 2024-08-09

**Authors:** David Amilo, Chinedu Izuchukwu, Khadijeh Sadri, Hao-Ren Yao, Evren Hincal, Yekini Shehu

**Affiliations:** 1 Mathematics Research Center, Near East University TRNC, Mersin 10, 99138 Nicosia, Turkey; 2 Department of Mathematics, Near East University TRNC, Mersin 10, 99138 Nicosia, Turkey; 3 Faculty of Art and Science, University of Kyrenia, Kyrenia, TRNC, Mersin 10, Kyrenia, Turkey; 4 School of Mathematics, University of the Witwatersrand, Private Bag 3, Johannesburg, 2050 South Africa; 5 National Institutes of Health, Bethesda, MD USA; 6 School of Mathematical Sciences, Zhejiang Normal University, Jinhua, 321004 People’s Republic of China

**Keywords:** Fractional-order model, Combination therapy, Heterogeneous lung cancer, Immunotherapy, Targeted therapy, Feedback controls, Optimization, Cancer, Computational biology and bioinformatics, Mathematics and computing

## Abstract

This research presents a novel approach to address the complexities of heterogeneous lung cancer dynamics through the development of a Fractional-Order Model. Focusing on the optimization of combination therapy, the model integrates immunotherapy and targeted therapy with the specific aim of minimizing side effects. Notably, our approach incorporates a clever fusion of Proportional-Integral-Derivative (PID) feedback controls alongside the optimization process. Unlike previous studies, our model incorporates essential equations accounting for the interaction between regular and mutated cancer cells, delineates the dynamics between immune cells and mutated cancer cells, enhances immune cell cytotoxic activity, and elucidates the influence of genetic mutations on the spread of cancer cells. This refined model offers a comprehensive understanding of lung cancer progression, providing a valuable tool for the development of personalized and effective treatment strategies. the findings underscore the potential of the optimized treatment strategy in achieving key therapeutic goals, including primary tumor control, metastasis limitation, immune response enhancement, and controlled genetic mutations. The dynamic and adaptive nature of the treatment approach, coupled with economic considerations and memory effects, positions the research at the forefront of advancing precision and personalized cancer therapeutics.

## Introduction

**Figure 1:**
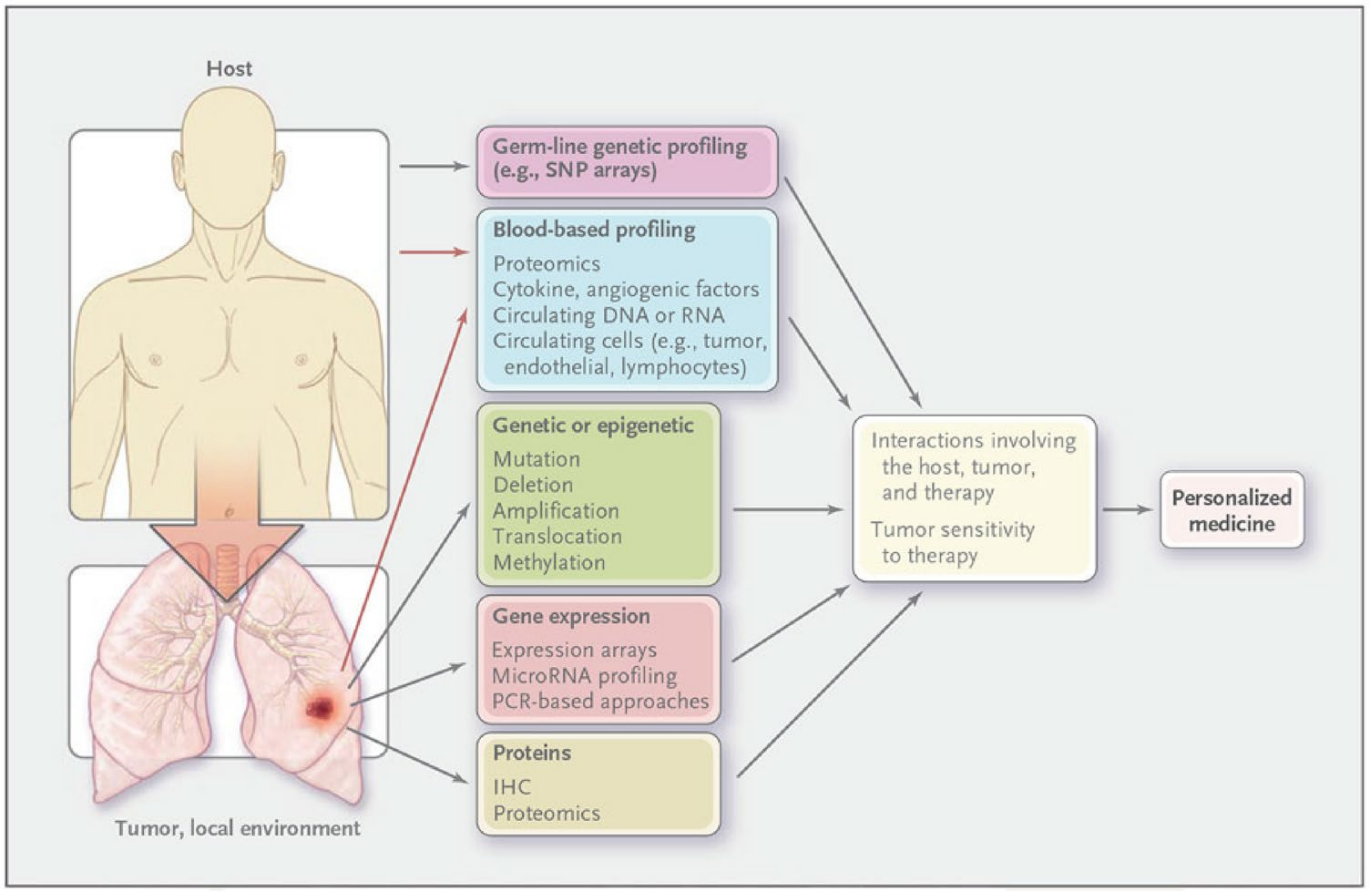
Lung Cancer Diagram^11^.

**Figure 2:**
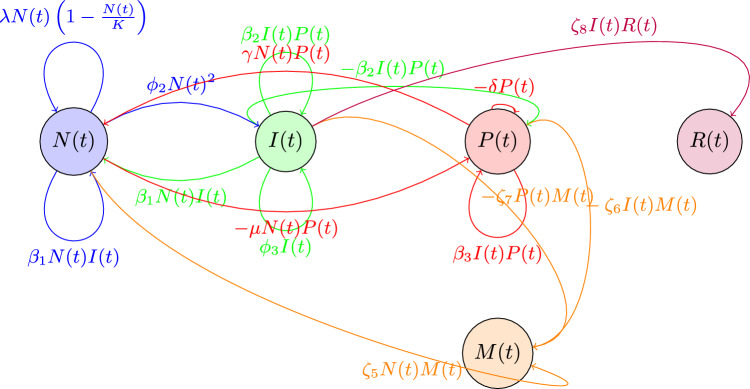
Schematic diagram of the lung cancer model in Eq. (1).

Lung cancer remains a serious challenge in oncology, and it continues to pose significant challenges owing to its heterogeneity and intricate dynamics^1,2^. Lung cancer, standing as the foremost cause of cancer-related mortality globally and in the United States^3,4^, encompasses two primary categories: non-small-cell lung cancer (NSCLC) and small-cell lung cancer. Despite progress in early detection and established treatments, NSCLC often presents in advanced stages with unfavorable prognoses, urging a more profound exploration of its molecular intricacies^5,6^. This underscores the pressing need for an enhanced understanding of the molecular underpinnings and evolution of the disease. Diverse factors contribute to the genesis of lung cancer, with smoking, exposure to environmental pollutants, and genetic predispositions being primary culprits^7–10^. NSCLC, comprising squamous-cell carcinoma, adenocarcinoma, and large-cell lung cancer, is associated with smoking, with adenocarcinoma predominantly affecting nonsmokers^11^. The complex etiology underscores the urgency for sophisticated modeling approaches that can capture the multifaceted nature of lung cancer progression and guide the optimization of therapeutic interventions. Traditionally, lung cancer treatment has relied on aggressive modalities such as chemotherapy and radiotherapy^12–14^. While these interventions have demonstrated some success in reducing tumor burden, their efficacy is often accompanied by a high toll on patients’ well-being^15–17^. Chemotherapy, characterized by its systemic nature, indiscriminately targets rapidly dividing cells, leading to widespread toxicity and a plethora of side effects^18,19^. Radiotherapy, though targeted, can result in collateral damage to surrounding healthy tissues^20,21^, further exacerbating the burden on patients. This landscape of high side effects and suboptimal success rates with conventional treatments underscores the critical need for novel therapeutic strategies. In recent years, the field of oncology has witnessed a paradigm shift with the advent of immunotherapy and targeted therapy^22–24^. These innovative approaches offer more intricate and targeted aggression on cancer cells, promising improved outcomes with significantly reduced side effects. Immunotherapy, a revolutionary development, leverages the body’s immune system to recognize and combat cancer cells^25,26^. By enhancing the inherent ability of the immune system to identify and destroy cancerous cells, immunotherapy minimizes harm to healthy tissues. Targeted therapy, on the other hand, focuses on specific molecular pathways involved in cancer growth, tailoring treatment to the individual’s genetic profile. This precision-oriented approach not only enhances efficacy but also mitigates the collateral damage seen in traditional treatments^27–29^. The research in Ref.^30^ explored potential synergies between emerging cancer treatment modalities—targeted therapies and cancer immunotherapies. Targeted therapies, designed to inhibit specific molecular pathways crucial for tumor growth, were observed to impact immune development and function. The study demonstrated the ability of targeted therapies to enhance processes like dendritic cell maturation, T cell priming, and the formation of enduring memory T cells. The authors suggested potential combinations of cancer vaccines with targeted therapies to amplify vaccine responses and improve effector T cell function. Furthermore, targeted therapies were found to sensitize tumor cells to immune-mediated killing by influencing the expression of death receptors and pro-survival signals. This enhanced immune-mediated tumor clearance suggested the potential of combining targeted therapies with immunotherapies for more efficient anti-tumor responses. Additionally, targeted therapies exhibited promise in reducing tumor-mediated immunosuppression by inhibiting tumorigenic inflammation and suppressing immunosuppressive cell types. This reduction in immunosuppression could potentially synergize with immunotherapies designed to generate anti-tumor T cells or enhance their effector function. The authors underscored the importance of strategically optimizing the dose, sequence, and timing of targeted therapies in designing future clinical trials. This approach aims to maximize anti-tumor efficacy while minimizing potential immunosuppressive side effects. Fractional calculus is an extension and generalization of integer calculus. Fractional calculus, with its memory property, proves advantageous in modeling and controlling various physical phenomena, showcasing its utility in closed-loop systems. Many research in recent times has employed the use of fractional calculus^31–38^. Authors of Ref.^39^ studied the suitability of fractional-order models in comparison with integer-order models in disease progressions. Their research underscored the advantages of fractional calculus, leveraging its memory property, a crucial aspect in characterizing biological processes. Their study proposes further exploration of fractional calculus in control theory, highlighting its utility in capturing characteristics evasive to integer order systems. Author of Ref.^40^ highlights the advantages of fractional-order modeling, considering memory trace and hereditary traits in cancer. They modeled the interactions between tumor cells, macrophages, active macrophages, and normal tissue cells, emphasizing the role of macrophages and normal cells in tumor growth and regression. Their findings demonstrate that host cell competitiveness and tumor growth rate significantly influence tumor cell loss and eradication time. The study suggests that fractional-order equations provide a more accurate explanation of cancer progression, particularly in capturing the different structural aspects of cancer. However, the research did not explicitly consider heterogeneity and include optimization and feedback controls for treatment regimes with a focus on reducing potential side effects. In Ref.^41^, the authors developed a Fractional Tumor-Immune Interaction Model for Lung Cancer (FTIIM-LC). The study employs the generalized Laguerre polynomials (GLPs) method to derive the optimal solution for the FTIIM-LC model. While the numerical simulation provides valuable insights, it may not comprehensively capture the intricacies of real-world tumor-immune interactions, underscoring the need for additional empirical validation. Furthermore, the study lacks explicit discussions on potential uncertainties or sensitivity analysis concerning model parameters, and critical considerations for real-world applications. Another author’s research^42^ focused on exploring the dynamics of lung cancer through a fractional-order mathematical model that examines the combined effects of surgery and immunotherapy. The study aims to optimize treatment dosage based on tumor response using a feedback control system designed with control theory, applying Pontryagin’s Maximum Principle to derive optimal conditions^34,43^. To better study the behavior of the proposed model, this model and its corresponding optimal system are solved using a predictor-corrector method from the Adams-Bashforth family^34^. To construct the proposed method, Lagrange interpolation method is used^44^. Numerical results indicate improved patient outcomes with the combined therapy, and the analysis emphasizes the sensitivity of the steady-state solution to specific parameters. The optimization models demonstrate improved treatment and dosage adjustments, reducing cancer growth. The incorporation of a Proportional-Integral-Derivative (PID) controller enhances the precision of dosage adjustments, maintaining the actual cancer cell population within a specified tolerance of the target. However, their model lacked considerations for genetic mutations and immune cells with enhanced cytotoxic activity. This omission impeded a thorough understanding of the complex dynamics involved in lung cancer progression. To address this limitation, our research introduces an improved model featuring essential equations. These additions capture the interaction between regular cancer cells and mutated cells, outline the dynamics between immune cells and mutated cancer cells, enhance immune cell cytotoxic activity, and elucidate the impact of genetic mutations on the spread of cancer cells. This enhanced model enhances our grasp of lung cancer dynamics, overcoming the constraints identified in their earlier study. Fractional-order modeling, employed in our research, offers distinct advantages over traditional integer-order models in capturing the complexities of lung cancer progression. Fractional-order derivatives introduce memory effects, allowing for a more accurate representation of dynamic systems with long-term dependencies^45,46^. This characteristic is particularly pertinent in modeling cancer dynamics, where intricate interactions unfold over extended periods. The fractional-order approach enables a more faithful representation of the underlying biological processes, enhancing the predictive power of the model. PID control, or Proportional-Integral-Derivative control, is a fundamental feedback mechanism widely used in engineering and automation^47,48^. It regulates systems by continuously adjusting inputs based on the difference between a desired setpoint and the current state. The Proportional Action responds to the current error, the Integral Action addresses accumulated error over time, and the Derivative Action anticipates future changes. PID control has diverse applications, including industrial automation, robotics, and medical processes like drug delivery and patient temperature regulation, due to its versatility and effectiveness in maintaining stability and achieving precise control. Our research builds upon this transformative shift, aiming to integrate the benefits of immunotherapy and targeted therapy within the framework of a fractional-order model. The goal is to optimize the combination of these therapies, maximizing their impact on cancer cells while minimizing potential side effects. The intricate interplay between genetic mutations, immune responses, and the spread of cancer cells within the proposed fractional-order model aligns seamlessly with the nuances of these advanced medical interventions. The novelty of our research lies in the integration of fractional-order modeling with the optimization of combination therapies for lung cancer. While previous models have explored the dynamics of cancer progression or focused on optimizing specific treatments, our approach uniquely combines these elements. By intricately incorporating immunotherapy and targeted therapy into a fractional-order framework, our research seeks to provide a comprehensive tool for tailoring combination therapies based on the specific characteristics of the patient’s cancer. Furthermore, our research introduces a novel dimension by integrating feedback control mechanisms, specifically PID controllers, into the optimization process. This addition enhances the adaptability of the model to dynamic changes in the cancer microenvironment, ensuring that the therapy remains effective throughout treatment. This dynamic approach sets our research apart, acknowledging the evolving nature of cancer and the need for personalized, adaptive interventions. In this transformative era of lung cancer treatment, our research aspires to contribute not only to the theoretical understanding of lung cancer dynamics but also to the practical realm of personalized medicine. By tailoring combination therapies through a fractional-order optimization model, we seek to amplify the positive impact of immunotherapy and targeted therapy, while reducing side effects, ushering in a new era of precision medicine in the heterogeneous landscape of lung cancer. Ultimately, our endeavor aims to advance the prospects for improved patient outcomes and a paradigm shift in the approach to lung cancer treatment. In therapeutic strategies, a paramount focus emerges on the integration of immunotherapy and targeted therapy^49–51^. Immunotherapy, strategically designed to fortify the body’s immune response against cancer, synergistically combines with targeted therapy to disrupt specific molecular pathways driving cancer growth. This holistic therapeutic integration lays the foundation for a comprehensive exploration of optimized treatment strategies within the context of fractional-order modeling. Within this dynamic landscape, an adaptive PID control strategy assumes a pivotal role in optimizing the administration of immunotherapy and targeted therapy. This sophisticated control mechanism dynamically adjusts drug dosages based on real-time error signals, ensuring a finely tuned and personalized approach to treatment. At the forefront of this strategy is the acknowledgment of inherent variability among patients, emphasizing the significance of personalized medicine. By tailoring therapeutic interventions to individual characteristics and responses, this approach seeks to maximize efficacy while minimizing adverse effects. The integration of fractional-order modeling and adaptive control strategies signifies a paradigm shift towards precision and personalized cancer therapeutics. Navigating the intricacies of cancer treatment optimization necessitates the incorporation of real-time patient data as a pivotal aspect of the model. This real-time patient data introduces additional variables and biomarkers into the model, facilitating a dynamic adaptation of the therapeutic strategy based on emerging patient-specific variables that influence treatment outcomes. Beyond the immediate treatment period, the consideration of long-term effects and survivorship dynamics broadens the scope of the study. This extended temporal perspective aims to assess the enduring impacts of the proposed therapeutic interventions on the patient’s well-being. Beyond the clinical realm, the economic implications of the proposed therapeutic strategy come into sharp focus. A meticulous cost-benefit analysis provides insights into the economic efficiency of the treatment, meticulously weighing direct costs against indirect costs and societal benefits. This economic perspective assumes a crucial role in guiding resource allocation and decision-making within healthcare systems. Additionally, the identification of memory effects within the model contributes biological realism to the computational framework. These memory effects, reflecting the persistent influence of past events on current states, seamlessly align with clinical observations and provide a more accurate representation of the dynamic interplay within the cancer microenvironment. The integration of economic considerations and the recognition of memory effects contribute to a more holistic and realistic approach to cancer modeling and treatment optimization. The proposed fractional-order model, is detailed in system (1) and further extended to system (10). It follows the governing principles of lung cancer dynamics as depicted in Fig. 1. The model in system (1) is illustrated in the schematic diagram in Fig. 2, and it incorporates variables and parameters described in Tables 1 and 2, respectively. Our approach offers several advantages over existing models. Unlike previous models that typically focus either on the dynamics of cancer progression or the optimization of specific treatments, our model uniquely combines these elements. By incorporating both immunotherapy and targeted therapy within a fractional-order framework, our model provides a more holistic view of treatment strategies. This allows for a more nuanced understanding of how different therapies can be tailored based on the specific characteristics of a patient’s cancer. The use of fractional-order calculus in our model offers a more flexible and accurate representation of the complex dynamics involved in cancer progression and treatment response. This mathematical approach captures the memory and hereditary properties of biological systems, which are often overlooked in integer-order models. Additionally, our research introduces the novel integration of PID (Proportional-Integral-Derivative) controllers into the optimization process. This feedback control mechanism enhances the model’s adaptability to dynamic changes in the cancer microenvironment. By continuously adjusting the therapy parameters, the PID controllers ensure that the treatment remains effective throughout the course of the therapy, accommodating the evolving nature of cancer. The combination of a fractional-order framework with feedback control mechanisms allows for highly personalized treatment plans. This dynamic approach acknowledges that cancer is not a static disease and requires interventions that can adapt to ongoing changes within the tumor and its environment. This is a significant advancement over traditional models, which often apply a one-size-fits-all strategy. However, our approach also has some limitations. The integration of fractional-order calculus and PID controllers increases the complexity of the model, which may result in higher computational demand. This can be a barrier to its implementation in clinical settings where quick decision-making is crucial. Our model requires detailed patient-specific data to accurately tailor the therapies. Collecting and validating this data can be challenging and resource-intensive. Furthermore, the model’s effectiveness is heavily dependent on the quality and accuracy of the input data. While our model shows promise in a theoretical and simulated environment, extensive clinical trials are necessary to validate its real-world applicability. Ensuring that the model performs well across diverse patient populations and cancer types remains a significant hurdle. The application of our approach requires close coordination between oncologists, data scientists, and control engineers. This interdisciplinary requirement can be challenging to achieve in practice, potentially limiting the widespread adoption of the model. Our paper is structured as follows: we establish fundamental concepts in Section "Preliminaries", introduce the model and establish existence and uniqueness in Section "Material and methods", conduct stability analysis in Section "Stability analysis", explore optimization with drug intervention in Section "Optimization", integrate feedback PID controls into the model in Section "Feedback control with PID controller", propose patient stratification and personalized medicine in Section Patient stratification and personalized medicine, conduct cost-benefit analysis in Section "Cost-benefit analysis", investigate long-term effects and survivorship in Section "Long-term effects and survivorship", and perform numerical analysis in Section Numerical analysis. Finally, we present our results and conclusions in Sections "Result and discussion" and "Conclusion", respectively.

**Table 1:** Variables of the Extended Lung Cancer Model.

Variable	Description
*N*(*t*)	Number of cancer cells in the lung tissue at time *t*.
*P*(*t*)	Number of cancer cells that have spread to other parts of the body at time *t*.
*I*(*t*)	Number of immune cells in the lung tissue at time *t*.
*M*(*t*)	Represents genetic mutations or subpopulations of cancer cells with different characteristics.
*R*(*t*)	Represents immune cells with enhanced cytotoxic activity.

**Table 2:** Parameters of the Extended Lung Cancer Model.

Parameter	Description
$\lambda ^{\alpha }$	Growth rate of cancer cells in the absence of constraints.
$K^{\alpha }$	Carrying capacity, representing the maximum sustainable population of cancer cells in the lung tissue.
$\mu ^{\alpha }$	Rate at which cancer cells are inhibited by the presence of immune cells.
$\beta ^{\alpha }_1$	Rate of interaction between cancer cells and immune cells.
$\phi ^{\alpha }_1, \phi ^{\alpha }_2, \phi ^{\alpha }_3$	Parameters related to the effects of growth factors on immune cells and cancer cells.
$\gamma ^{\alpha }$	Rate of growth of cancer cells that have spread to other parts of the body.
$\delta ^{\alpha }$	Rate at which cancer cells that have spread die off.
$\beta ^{\alpha }_2$	Rate of interaction between immune cells and cancer cells in the lung tissue.
$\beta ^{\alpha }_3$	Rate of interaction between blood vessels and cancer cells, affecting the spread of cancer.
$\zeta ^{\alpha }_1$ to $\zeta ^{\alpha }_8$	Parameters controlling rates of genetic mutations, interaction rates, and immune enhancement rates.

## Preliminaries

In this section, we recall some definitions and properties of fractional integral and derivative, which will be used later.

### Definition 2.1

Let $\alpha \in {\mathbb {R}}, n-1< \alpha \le n, n \in {\mathbb {N}},$ and *g*(*t*) is an absolutely continuous function on the interval $[0, \infty )$, then the Caputo fractional derivative of order $\alpha$ is defined as Refs.^52,53^:

$$\begin{aligned} _{0}^{c}D_{t}^{\alpha } g(t)=\frac{1}{\Gamma (n-\alpha )} \int _0^t (t-s)^{n-\alpha -1} \, g^{(n)}(s)\, ds, \quad t\in [0, T_f], \,\, \alpha \in (0, 1], \end{aligned}$$

where *g*(*t*) is an *n* times differentiable function and $\Gamma (x)$ is the Gamma function given as follows:

$$\begin{aligned} \Gamma (x)=\int _0^ {\infty } e^{-z} \, z^{x-1}\, dz, \quad Re(z)>0. \end{aligned}$$

### Definition 2.2

The Riemann–Liouville fractional integral of order $\alpha >0$ of a function *g*(*t*) is defined as^52,53^:

$$\begin{aligned} _{0}^{RL}I_{t}^{\alpha } g(t)=\frac{1}{\Gamma (\alpha )} \int _0^t (t-s)^{\alpha -1} \, g(s)\, ds, \quad t\in [0, T_f]. \end{aligned}$$

The above integral exists almost everywhere for any integrable function *g*(*t*).

The Riemann–Liouville integral and the Caputo fractional derivative operators satisfy the following property:

$$\begin{aligned} _{0}^{RL}I_{t}^{\alpha } ( _{0}^{c}D_{t}^{\alpha } g(t)) =g(t)-\sum \limits _{k=0}^{n-1} g^{(k)}(0)\frac{t^k}{k!}, \quad n-1<\alpha \le n. \end{aligned}$$

## Material and methods

### The model

1 $$\begin{aligned} _{0}^{c}D_{t}^{\alpha }N(t)&= \lambda ^{\alpha }N(t)\left( 1 - \frac{N(t)}{K^{\alpha }}\right) - \mu ^{\alpha } N(t)P(t) - \beta ^{\alpha }_1N(t)I(t) - \zeta ^{\alpha }_1 N(t)M(t),\\ _{0}^{c}D_{t}^{\alpha }I(t)&= \phi ^{\alpha }_1I_0 + \phi ^{\alpha }_2N(t)^2 - \phi ^{\alpha }_3I(t) - \beta ^{\alpha }_2I(t)P(t) + \zeta ^{\alpha }_2 I(t)M(t) - \zeta ^{\alpha }_3 I(t)R(t),\\ _{0}^{c}D_{t}^{\alpha }P(t)&= \gamma ^{\alpha } N(t)P(t) - \delta ^{\alpha } P(t) - \beta ^{\alpha }_3I(t)P(t) + \zeta ^{\alpha }_4 P(t)M(t),\\ _{0}^{c}D_{t}^{\alpha }M(t)&= \zeta ^{\alpha }_5 N(t)M(t) - \zeta ^{\alpha }_6 I(t)M(t) - \zeta ^{\alpha }_7 P(t)M(t),\\ _{0}^{c}D_{t}^{\alpha }R(t)&= \zeta ^{\alpha }_8 I(t)R(t), \end{aligned}$$

with the following initial conditions:

$$\begin{aligned} N(0)&=N_0>0, \quad I(0)=I_0>0, \quad P(0)=P_0>0, \\ M(0)&=M_0>0, \quad R(0)=R_0>0, \end{aligned}$$

where $_{0}^{c}D_{t}^{\alpha }$ is the Caputo fractional differential operator. All variables and parameters in system (1) are non-negative.

Explanation of terms in Eq. (1) are as follows:

1. Equation for *N*(*t*) (Number of Cancer Cells):
  $$\begin{aligned} \lambda ^{\alpha }N(t)\left( 1 - \frac{N(t)}{K^{\alpha }}\right)&: \text {Logistic growth term with carrying capacity }K^{\alpha }.\\ - \mu ^{\alpha } N(t)P(t)&: \text {Inhibition of cancer cell growth by immune cells.}\\ - \beta ^{\alpha }_1N(t)I(t)&: \text {Interaction between cancer cells and immune cells.}\\ - \zeta ^{\alpha }_1 N(t)M(t)&: \text {Incorporation of genetic mutations affecting cancer cell dynamics.} \end{aligned}$$
2. Equation for *I*(*t*) (Number of Immune Cells):
  $$\begin{aligned} \phi ^{\alpha }_1I_0 + \phi ^{\alpha }_2N(t)^2&: \text {Growth of immune cells influenced by growth factors and cancer cell concentration.}\\ - \phi ^{\alpha }_3I(t)&: \text {Inhibition of immune cell growth.}\\ - \beta ^{\alpha }_2I(t)P(t)&: \text {Interaction between immune cells and cancer cells.}\\ + \zeta ^{\alpha }_2 I(t)M(t)&: \text {Interaction between immune cells and mutated cancer cells.}\\ - \zeta ^{\alpha }_3 I(t)R(t)&: \text {Enhanced cytotoxic activity of immune cells.} \end{aligned}$$
3. Equation for *P*(*t*) (Number of Cancer Cells that Spread):
  $$\begin{aligned} \gamma ^{\alpha } N(t)P(t)&: \text {Growth of cancer cells that have spread.}\\ - \delta ^{\alpha } P(t)&: \text {Death of spread cancer cells.}\\ - \beta ^{\alpha }_3I(t)P(t)&: \text {Interaction between immune cells and spread cancer cells.}\\ + \zeta ^{\alpha }_4 P(t)M(t)&: \text {Influence of genetic mutations on spread cancer cells.} \end{aligned}$$
4. Equation for *M*(*t*) (Genetic Mutations):
  $$\begin{aligned} \zeta ^{\alpha }_5 N(t)M(t)&: \text {Interaction between regular cancer cells and mutated cells.}\\ - \zeta ^{\alpha }_6 I(t)M(t)&: \text {Interaction between immune cells and mutated cancer cells.}\\ - \zeta ^{\alpha }_7 P(t)M(t)&: \text {Influence of genetic mutations on spread cancer cells.} \end{aligned}$$
5. Equation for *R*(*t*) (Enhanced Immune Cells):
  $$\begin{aligned} \zeta ^{\alpha }_8 I(t)R(t): \text {Enhancement of immune cell cytotoxic activity.} \end{aligned}$$

The proposed model in Eq. (1) provides a comprehensive framework for understanding the dynamic complexities of lung cancer progression, integrating real-life scenarios through a system of fractional-order differential equations. The five variables, $N(t)$ (cancer cells), $I(t)$ (immune cells), $P(t)$ (spread cancer cells), $M(t)$ (genetic mutations), and $R(t)$ (enhanced immune cells), interact in an intricate manner that mirrors the intricate dynamics observed in actual lung cancer cases. The equation for $N(t)$ captures the growth and inhibition of cancer cells, influenced by immune responses and genetic mutations. The logistic growth term with a carrying capacity ($K^{\alpha }$) reflects the limitations on cancer cell proliferation, mirroring real cases where the availability of resources imposes constraints. The interaction terms with immune cells ($I(t)$) and genetic mutations ($M(t)$) illustrate the multifaceted nature of the immune response and the impact of genetic alterations on cancer cell dynamics. In the equation for $I(t)$, the growth of immune cells is influenced by growth factors and the concentration of cancer cells. This mirrors actuality where immune responses are stimulated by the presence of cancer cells and other growth factors. The inhibition term reflects the natural regulatory mechanisms controlling immune cell proliferation. The interaction terms with cancer cells ($N(t)$) and mutated cells ($M(t)$) depict the immune response’s intricate role in recognizing and interacting with both regular and mutated cancer cells. The spread of cancer cells ($P(t)$) is governed by factors such as growth, death, and interactions with immune cells. This mimics the real-life scenario where cancer cells may undergo metastasis, with the immune system playing a role in controlling or influencing this process. The influence of genetic mutations ($M(t)$) on the spread of cancer cells highlights the genetic heterogeneity observed in lung cancer and its impact on disease progression. The dynamics of genetic mutations ($M(t)$) involve interactions between regular and mutated cancer cells, reflecting the genomic instability observed in actual lung cancer cases. The influence of genetic mutations on the spread of cancer cells ($P(t)$) underscores the role of genetic alterations in driving the spread and aggressiveness of the disease. The enhancement of immune cell cytotoxic activity ($R(t)$) reflects actuality where the immune system adapts to recognize and target cancer cells more effectively. The parameters associated with these equations, such as growth rates, interaction strengths, and mutation rates, are carefully chosen to mirror the physiological characteristics of lung cancer progression. The model operates under the influence of the Caputo fractional differential operator, introducing memory effects to capture the persistence of interactions over time. This aligns with the real-life scenario where past interactions influence the current state of the system. The initialization of the model with non-negative values for variables corresponds to the physiological fact that cell populations cannot be negative. These equations provide a detailed representation of the fractional-order lung cancer model, capturing the intricate dynamics involving genetic mutations, immune responses, and the spread of cancer cells. The fractional-order derivatives add a detailed dimension to the model, allowing for a more accurate representation of the complex interactions within the lung cancer system. In summary, the fractional-order lung cancer model intricately encapsulates the interplay of various factors observed in actual lung cancer cases. From the constraints imposed by resource availability to the intricate interactions between immune responses and cancer cells, the model provides a comprehensive framework for studying the dynamic and heterogeneous nature of lung cancer progression. 

### Existence and uniqueness of the solution

We rewrite system (1) as:

2 $$\begin{aligned} ^{c}_{0}D^{\alpha }_t X(t)=B_1 X(t)+N(t)B_2 X(t)+I(t) B_3 X(t)+P(t)B_4 X(t)+M(t) B_5 X(t),~~X(0)=X_0, \end{aligned}$$

where,

$$\begin{aligned} X(t)= & {} \begin{pmatrix} N(t)\\ I(t)\\ P(t)\\ M(t)\\ R(t) \end{pmatrix}, ~~~B_1=\begin{pmatrix} \lambda ^{\alpha }&{}0&{}0&{}0&{}0\\ 0&{}-\phi ^{\alpha }_3&{}0&{}0&{}0\\ 0&{}0&{}-\delta ^{\alpha }&{}0&{}0\\ 0&{}0&{}0&{}0&{}0\\ 0&{}0&{}0&{}0&{}0\\ \end{pmatrix},~~~~B_2=\begin{pmatrix} \frac{-\lambda ^{\alpha }}{K^{\alpha }} &{}-\beta ^{\alpha }_1 &{}-\mu ^{\alpha } &{}-\zeta ^{\alpha }_1 &{} 0\\ \phi _2^{\alpha }&{}0&{}0&{}0&{}0\\ 0&{}0&{}\gamma ^{\alpha }&{}0&{}0\\ 0&{}0&{}0&{}0&{}0\\ 0&{}0&{}0&{}0&{}0\\ \end{pmatrix},\\ B_3= & {} \begin{pmatrix} 0&{}0&{}0&{}0&{}0\\ 0&{}0&{}-\beta ^{\alpha }_2&{}\zeta ^{\alpha }_2&{}-\zeta ^{\alpha }_3\\ 0&{}0&{}-\beta ^{\alpha }_3&{}0&{}0\\ 0&{}0&{}0&{}-\zeta ^{\alpha }_6&{}0\\ 0&{}0&{}0&{}0&{}\zeta ^{\alpha }_8\\ \end{pmatrix},~~~~B_4=\begin{pmatrix} -\mu ^{\alpha }&{}0&{}0&{}0&{}0\\ 0&{}0&{}0&{}0&{}0\\ \gamma ^{\alpha }&{}0&{}0&{}\zeta ^{\alpha }_4&{}0\\ 0&{}0&{}0&{}-\zeta ^{\alpha }_7&{}0\\ 0&{}0&{}0&{}0&{}0\\ \end{pmatrix},~~~~B_5=\begin{pmatrix} 0&{}0&{}0&{}0&{}0\\ 0&{}0&{}0&{}0&{}0\\ 0&{}0&{}\zeta ^{\alpha }_4&{}0&{}0\\ \zeta ^{\alpha }_5&{}0&{}-\zeta ^{\alpha }_7&{}0&{}0\\ 0&{}0&{}0&{}0&{}0\\ \end{pmatrix}. \end{aligned}$$

#### Theorem 3.1

$X\in C^*[0,\tau ]$ is the unique solution of the system (2).

#### Proof

By the Riemann-Liouville fractional integral 2.2 we obtain:

3 $$\begin{aligned} X(t)&=X(0) +\frac{1}{\Gamma (\alpha )}\int _{0}^{t}(t-s)^{\alpha -1}\Big (B_1 X(s)+N(s)B_2 X(s)+I(s) B_3 X(s)\\&\quad +P(s)B_4 X(s)+M(s) B_5 X(s)\Big )ds. \end{aligned}$$

Now, let us define $T:C^*[0,\tau ]\rightarrow C^*[0,\tau ]$ by:

$$\begin{aligned} TX(t)= & {} X_0+\frac{1}{\Gamma (\alpha )}\int _{0}^{t}(t-s)^{\alpha -1}\Big (B_1 X(s)+N(s)B_2 X(s)+I(s) B_3 X(s)\\{} & {} +P(s)B_4 X(s)+M(s) B_5 X(s)\Big )ds. \end{aligned}$$

Then, we have:

$$\begin{aligned} e^{-Lt}(TX-TY)= & {} e^{-Lt}\Big [\frac{1}{\Gamma (\alpha )}\int _{0}^{t}(t-s)^{\alpha -1}\Big (B_1 \Big (X(s)-Y(s)\Big )\\{} & {} +N(t)B_2 \Big (X(s)-Y(s)\Big )+I(s) B_3 \Big (X(s)-Y(s)\Big )\\{} & {} +P(t)B_4 \Big (X(s)-Y(s)\Big )+M(s) B_5 \Big (X(s)-Y(s)\Big )\Big )\Big ]\\\le & {} \frac{1}{\Gamma (\alpha )}\int _{0}^{t}(t-s)^{\alpha -1}e^{-L(t-s)}\Big (X(s)-Y(s)\Big )\\{} & {} \times e^{-Ls}\Big (B_1+pB_2+rB_3+sB_4+vB_5\Big )ds, \end{aligned}$$

where *p*, *r*, *s*, and *v* are the maximum values of *N*(*t*), *I*(*t*), *P*(*t*), and *M*(*t*). Thus,

$$\begin{aligned} \Vert e^{-Lt}(TX-TY)\Vert \le \Big |L^{-\alpha }\Big (B_1+pB_2+rB_3+sB_4+vB_5\Big )\Big | \, \Vert X-Y\Vert \, \Big \Vert \frac{1}{\Gamma (\alpha )}\int _{0}^{t}s^{\alpha -1} ds \Big \Vert . \end{aligned}$$

Hence,

$$\begin{aligned} \Vert TX-TY\Vert \le \frac{\tau ^{\alpha }}{\Gamma (\alpha +1)}\Big |L^{-\alpha }\Big (B_1+pB_2+rB_3+sB_4+vB_5\Big )\Big | \,\Vert X-Y\Vert . \end{aligned}$$

Now, if we choose *L* such that $\Vert L^\alpha \Vert >\frac{\tau ^{\alpha }}{\Gamma (\alpha +1)} |B_1+pB_2+rB_3+sB_4+vB_5|$, then,

$$\begin{aligned} \Vert TX-TY\Vert \le \kappa \Vert X-Y\Vert , \end{aligned}$$

where $\kappa = \frac{\tau ^{\alpha }}{\Gamma (\alpha +1)}\Big |L^{-\alpha }\Big (B_1+pB_2+rB_3+sB_4+vB_5\Big )\Big |<1.$ Therefore, *T* is a contraction and by the Banach contraction mapping principle, *T* has a unique fixed point. That is (3) has a unique solution $X\in C^*[0, \tau ]$. Since (3) is equivalent to the Volterra integral equation that is equivalent to system (2), we can conclude that $X\in C^*[0, \tau ]$ is the unique solution of system (2). $\square$

## Stability analysis

### Equilibrium points

To obtain the equilibrium points of system (1), we proceed as follows:

$$\begin{aligned} _{0}^{c}D_{t}^{\alpha }N(t) = {_{0}^{c}D_{t}^{\alpha }}I(t)= {_{0}^{c}D_{t}^{\alpha }}P(t)= {_{0}^{c}D_{t}^{\alpha }}M(t)= {_{0}^{c}D_{t}^{\alpha }}R(t)=0. \end{aligned}$$

That is, we set the system to zero and solve simultaneously.

4 $$\begin{aligned}&\lambda ^{\alpha }N(t)\left( 1 - \frac{N(t)}{K^{\alpha }}\right) - \mu ^{\alpha } N(t)P(t) - \beta ^{\alpha }_1N(t)I(t) - \zeta ^{\alpha }_1 N(t)M(t)=0, \end{aligned}$$

5 $$\begin{aligned}&\phi ^{\alpha }_1I_0 + \phi ^{\alpha }_2N(t)^2 - \phi ^{\alpha }_3I(t) - \beta ^{\alpha }_2I(t)P(t) + \zeta ^{\alpha }_2 I(t)M(t) - \zeta ^{\alpha }_3 I(t)R(t)=0, \end{aligned}$$

6 $$\begin{aligned}&\gamma ^{\alpha } N(t)P(t) - \delta ^{\alpha } P(t) - \beta ^{\alpha }_3I(t)P(t) + \zeta ^{\alpha }_4 P(t)M(t)=0, \end{aligned}$$

7 $$\begin{aligned}&\zeta ^{\alpha }_5 N(t)M(t) - \zeta ^{\alpha }_6 I(t)M(t) - \zeta ^{\alpha }_7 P(t)M(t)=0, \end{aligned}$$

8 $$\begin{aligned}&\zeta ^{\alpha }_8 I(t)R(t)=0. \end{aligned}$$

The computation for the search for the equilibrium points is very complicated due to the structure of our model. Hence, we will not include the computational steps here, but list the equilibrium points that we obtained, as follows:

$$E_0=(0,0,0,0,0)$$,

$$E_1=\left(0,0,0,\frac{\delta ^\alpha }{\zeta _4^\alpha },0\right)$$,

$$E_2=\left(0,\frac{\phi ^\alpha _1 I_0}{\phi _3^\alpha },0,0,0\right)$$,

$$E_3=\left(0,\frac{-\delta ^\alpha }{\beta _3^\alpha },\frac{-\beta _3^\alpha \phi ^\alpha _1I_0+\phi ^\alpha _3\delta ^\alpha }{\beta _2^\alpha \delta ^\alpha },0,0\right)$$,

$$E_4=\left(0,\frac{\zeta _4^\alpha M^*-\delta ^\alpha }{\beta _3^\alpha }, \frac{\zeta _6^\alpha (\delta ^\alpha -\zeta _4^\alpha M^*)}{\beta _3^\alpha \zeta _7^\alpha },M^*,0\right)$$,

$$E_5=\left(0,\frac{\zeta _4^\alpha M^{**}-\delta ^\alpha }{\beta _3^\alpha }, \frac{\zeta _6^\alpha (\delta ^\alpha -\zeta _4^\alpha M^{**})}{\beta _3^\alpha \zeta _7^\alpha },M^{**},0\right)$$,

where

$$M^*=\frac{-b+\sqrt{b^2-4ac}}{2a}$$ and $$M^{**}=\frac{-b-\sqrt{b^2-4ac}}{2a}$$,

$$a=(\beta _3^\alpha )^2\zeta _2^\alpha \zeta _4^\alpha \zeta _7^\alpha \zeta _6^\alpha + \beta _3^\alpha \beta _2^\alpha (\zeta _4^\alpha )^2(\zeta _6^\alpha )^2$$,

$$b=-((\beta _3^\alpha )^2\zeta _7^\alpha \zeta _2^\alpha \zeta _6^\alpha \delta ^\alpha +(\beta _3^\alpha )^2\zeta _7^\alpha \phi _3^\alpha \zeta _6^\alpha \zeta _4^\alpha +2\beta _2^\alpha \beta _3^\alpha (\zeta _3^\alpha )^2\delta ^\alpha \zeta _4^\alpha )$$,

$$c=(\beta _3^\alpha )^3\zeta _6^\alpha \zeta _7^\alpha \phi _1^\alpha I_0+(\beta _3^\alpha )^3\zeta _7^\alpha \zeta _6^\alpha \delta ^\alpha \phi _7^\alpha +\beta _3^\alpha \beta _2^\alpha (\zeta _6^\alpha )^2(\delta ^\alpha )^2$$,

$$E_6=\left(K^\alpha ,0,0,0,0\right)$$,

$$E_7=\left(\frac{\delta ^\alpha }{\gamma ^\alpha },0,\frac{\gamma ^\alpha \lambda ^\alpha K^\alpha -\delta ^\alpha \lambda ^\alpha }{\mu ^\alpha \gamma ^\alpha K^\alpha },0,0\right)$$,

$$E_8=\left(\frac{\delta ^\alpha }{\gamma ^\alpha },0,\frac{\gamma ^\alpha \lambda ^\alpha K^\alpha -\delta ^\alpha \lambda ^\alpha }{\mu ^\alpha \gamma ^\alpha K^\alpha },0,0\right)$$,

$$E_9=(\frac{\lambda ^\alpha K^\alpha \zeta _4^\alpha \zeta _7^\alpha -\lambda ^\alpha \zeta _1^\alpha \zeta _7^\alpha \delta ^\alpha }{\lambda ^\alpha \zeta _4^\alpha \zeta _7^\alpha +\mu ^\alpha K^\alpha \zeta _4^\alpha \zeta _5^\alpha -\lambda ^\alpha \zeta _1^\alpha \zeta _7^\alpha \gamma ^\alpha },0,\frac{\lambda ^\alpha K^\alpha \zeta _4^\alpha \zeta _5^\alpha \zeta _7^\alpha -\lambda ^\alpha \zeta _1^\alpha \zeta _5^\alpha \zeta _7^\alpha \delta ^\alpha }{\lambda ^\alpha \zeta _4^\alpha (\zeta _7^\alpha )^2+\mu ^\alpha K^\alpha \zeta _4^\alpha \zeta _5^\alpha \zeta _7^\alpha -\lambda ^\alpha \zeta _1^\alpha (\zeta _7^\alpha )^2\gamma ^\alpha },$$ $$\frac{\delta ^\alpha }{\zeta _4^\alpha }-\frac{\gamma ^\alpha \lambda ^\alpha K^\alpha \zeta _4^\alpha \zeta _7^\alpha -\lambda ^\alpha \zeta _1^\alpha \zeta _7^\alpha \delta ^\alpha \gamma ^\alpha }{\lambda ^\alpha (\zeta _4^\alpha )^2 \zeta _7^\alpha +\mu ^\alpha K^\alpha (\zeta _4^\alpha )^2 \zeta _5^\alpha -\lambda ^\alpha \zeta _1^\alpha \zeta _4^\alpha \zeta _7^\alpha \gamma ^\alpha },0)$$,

$$E_{10}=(\frac{-\phi _3^\alpha \lambda ^\alpha -\sqrt{(\phi _3^\alpha )^2(\lambda ^\alpha )^2+4K^\alpha \beta _1^\alpha \phi _2^\alpha (\phi _3^\alpha \lambda ^\alpha K^\alpha -\beta _1^\alpha K^\alpha \phi _1^\alpha I_0)}}{2K^\alpha \beta _1^\alpha \phi _2^\alpha },$$ $$\frac{(\lambda ^\alpha )^2 \phi _3^\alpha +2\phi _2^\alpha \lambda ^\alpha (K^\alpha )^2\beta _1^\alpha -\sqrt{((\lambda ^\alpha )^2 \phi _3^\alpha +2\phi _2^\alpha \lambda ^\alpha (K^\alpha )^2\beta _1^\alpha )^2-4\phi _2^\alpha (\beta _1^\alpha )^2(K^\alpha )^2(\phi _2^\alpha (\lambda ^\alpha )^2(K^\alpha )^2+(\lambda ^\alpha )^2\phi _1^\alpha I_0)}}{2\phi _2^\alpha (\beta _1^\alpha )^2(K^\alpha )^2},0,0, 0)$$,

$$E_{11}=(\frac{-\phi _3^\alpha \lambda ^\alpha +\sqrt{(\phi _3^\alpha )^2(\lambda ^\alpha )^2+4K^\alpha \beta _1^\alpha \phi _2^\alpha (\phi _3^\alpha \lambda ^\alpha K^\alpha -\beta _1^\alpha K^\alpha \phi _1^\alpha I_0)}}{2K^\alpha \beta _1^\alpha \phi _2^\alpha },$$ $$\frac{(\lambda ^\alpha )^2 \phi _3^\alpha +2\phi _2^\alpha \lambda ^\alpha (K^\alpha )^2\beta _1^\alpha +\sqrt{((\lambda ^\alpha )^2 \phi _3^\alpha +2\phi _2^\alpha \lambda ^\alpha (K^\alpha )^2\beta _1^\alpha )^2-4\phi _2^\alpha (\beta _1^\alpha )^2(K^\alpha )^2(\phi _2^\alpha (\lambda ^\alpha )^2(K^\alpha )^2+(\lambda ^\alpha )^2\phi _1^\alpha I_0)}}{2\phi _2^\alpha (\beta _1^\alpha )^2(K^\alpha )^2},0,0, 0)$$,

$$E_{12}=\left(N^*,\frac{\gamma ^\alpha N^{*}-\delta ^\alpha }{\beta _3^\alpha }, \frac{\beta _3^\alpha \phi _1^\alpha I_0+\beta _3^\alpha \phi _2^\alpha (N^{*})^2-\phi _3^\alpha \gamma ^\alpha N^*+\phi _3^\alpha \delta ^\alpha }{\beta _2^\alpha \gamma ^\alpha N^*-\beta _2^\alpha \delta ^\alpha }, 0,0\right)$$,

$$E_{13}=\left(N^{**},\frac{\gamma ^\alpha N^{**}-\delta ^\alpha }{\beta _3^\alpha }, \frac{\beta _3^\alpha \phi _1^\alpha I_0+\beta _3^\alpha \phi _2^\alpha (N^{**})^2-\phi _3^\alpha \gamma ^\alpha N^{**}+\phi _3^\alpha \delta ^\alpha }{\beta _2^\alpha \gamma ^\alpha N^{**}-\beta _2^\alpha \delta ^\alpha }, 0,0\right)$$,

where

$$N^*=\frac{-b^*+\sqrt{b^{*2}-4a^*c^*}}{2a^*}$$ and $$N^{**}=\frac{-b^*-\sqrt{b^{*2}-4a^*c^*}}{2a^*}$$,

$$a^*=\beta _2^\alpha \beta _3^\alpha \gamma ^\alpha \lambda ^\alpha +\mu ^\alpha K^\alpha \phi _2^\alpha (\beta _3^\alpha )^2+\beta _2^\alpha \beta _1^\alpha K^\alpha (\gamma ^\alpha )^2$$,

$$b^*=\beta _2^\alpha \beta _3^\alpha \gamma ^\alpha \lambda ^\alpha K^\alpha +\beta _2^\alpha \beta _3^\alpha \delta ^\alpha \lambda ^\alpha +\mu ^\alpha K^\alpha \phi _3^\alpha \gamma ^\alpha +2\beta _1^\alpha \beta _2^\alpha \gamma ^\alpha K^\alpha \delta ^\alpha$$,

$$c^*=\beta _2^\alpha \beta _3^\alpha \lambda ^\alpha K^\alpha \delta ^\alpha +(\beta _3^\alpha )^2 \mu ^\alpha K^\alpha \phi _1^\alpha I_0 +\mu ^\alpha K^\alpha \phi _3^\alpha \delta ^\alpha -\beta _1^\alpha \beta _2^\alpha (\delta ^\alpha )^2 K^\alpha$$,

$$E_{14}=(\bar{N}, \bar{I}, \bar{P}, \bar{M}, \bar{R}),$$

where,

$$\bar{N}=\frac{\zeta ^\alpha _4(\zeta ^\alpha _6)^2\lambda ^\alpha K^\alpha -\zeta _1^\alpha \zeta _6^\alpha \delta ^\alpha \lambda ^\alpha -\left( (\zeta _6^\alpha )^2\zeta _4^\alpha \mu ^\alpha K^\alpha -\zeta _4^\alpha \zeta _6^\alpha \zeta _7^\alpha \beta _1^\alpha K^\alpha -\zeta _1^\alpha \zeta _7^\alpha \beta _3^\alpha \lambda ^\alpha \right) {\bar{P}}}{\zeta _4^\alpha (\zeta _6^\alpha )^2\lambda ^\alpha +\zeta _4^\alpha \zeta _5^\alpha \zeta _6^\alpha \beta _1^\alpha K^\alpha -\zeta _1^\alpha \zeta _6^\alpha \lambda ^\alpha \gamma ^\alpha +\zeta _1^\alpha \zeta _5^\alpha \beta _3^\alpha \lambda ^\alpha }$$,

$$\bar{I}= \frac{\zeta _4^\alpha \zeta _5^\alpha (\zeta _6^\alpha )^2\lambda ^\alpha K^\alpha -\zeta _1^\alpha \zeta _5^\alpha \zeta _6^\alpha \lambda ^\alpha -\left( \zeta _4^\alpha \zeta _5^\alpha (\zeta _6^\alpha )^2\mu ^\alpha K^\alpha -\zeta _1^\alpha \zeta _5^\alpha \zeta _7^\alpha \beta _3^\alpha \lambda ^\alpha +\zeta _4^\alpha (\zeta _6^\alpha )^2 \zeta _7^\alpha -\zeta _1^\alpha \zeta _6^\alpha \zeta _7^\alpha \lambda ^\alpha \gamma ^\alpha +\zeta _1^\alpha \zeta _5^\alpha \zeta _7^\alpha \beta _3^\alpha \lambda ^\alpha \right) {\bar{P}}}{\zeta _4^\alpha (\zeta _6^\alpha )^3\lambda ^\alpha +\zeta _4^\alpha \zeta _5^\alpha (\zeta _6^\alpha )^2\beta _1^\alpha K^\alpha -\zeta _1^\alpha (\zeta _6^\alpha )^2 \lambda ^\alpha \gamma ^\alpha +\zeta _1^\alpha \zeta _5^\alpha \zeta _6^\alpha \beta _3^\alpha \lambda ^\alpha }$$,

$$\bar{P}=\frac{-{\bar{b}}\pm \sqrt{\bar{b}^2-4(\zeta _6^\alpha \zeta _7^\alpha \beta _3^\alpha +\zeta _2^\alpha (\zeta _7^\alpha )^2\beta _3^\alpha )(\zeta _4^\alpha (\zeta _6^\alpha )^2\phi _1^\alpha I_0+\zeta _4^\alpha (\zeta _6^\alpha )^2\phi _2^\alpha -\zeta _4^\alpha \zeta _5^\alpha \zeta _6^\alpha \phi _3^\alpha +\zeta _2^\alpha \zeta _5^\alpha \beta _3^\alpha -\zeta _2^\alpha \zeta _5^\alpha \zeta _6^\alpha \gamma ^\alpha -\zeta _3^\alpha \zeta _5^\alpha \zeta _6^\alpha )}}{2(\zeta _6^\alpha \zeta _7^\alpha \beta _3^\alpha +\zeta _2^\alpha (\zeta _7^\alpha )^2\beta _3^\alpha )},$$ with $${\bar{b}}=\zeta _4^\alpha \zeta _6^\alpha \zeta _7^\alpha \phi _3^\alpha -\zeta _5^\alpha \zeta _6^\alpha \beta _3^\alpha -\zeta _2^\alpha \zeta _5^\alpha \zeta _7^\alpha \beta _3^\alpha -2\zeta _2^\alpha \zeta _5^\alpha \zeta _7^\alpha \beta _3^\alpha +\zeta _2^\alpha \zeta _6^\alpha \zeta _7^\alpha \gamma ^\alpha -\zeta _2^\alpha \zeta _6^\alpha \zeta _7^\alpha \delta ^\alpha +\zeta _3^\alpha \zeta _6^\alpha \zeta _7^\alpha$$,

$$\bar{M}=\frac{\beta _3^\alpha {\bar{I}}-\gamma ^\alpha +\delta ^\alpha }{\zeta _4^\alpha }$$,

$\bar{R}=\frac{\phi _1^\alpha I_0+\phi ^\alpha _2 N^2-(\phi _3^\alpha +\beta _2^\alpha {\bar{P}}-\zeta _2^\alpha {\bar{M}}){\bar{I}}}{\zeta _3^\alpha {\bar{I}}}$. Next, we establish the stability conditions of these equilibrium points. However, our primary aim is to focus on the full-blown cancer-immune dynamic case and look at those conditions for which the patient can survive, with regards to treatment modality and reduction of side effects^54^. Thus, in what follows, we shall only study the stability of the equilibrium point $E_{14}$ and omit the rest.

### Local stability

We now study the local stability of the endemic equilibrium point $E_{14}=(\bar{N}, \bar{I}, \bar{P}, \bar{M}, \bar{R})$. To do this, we have the following Jacobian matrix of system (1) $J(E_{14})$ computed at equilibrium point $E_{14}$:

$$\begin{aligned} \begin{pmatrix} \lambda ^{\alpha } - \frac{2\lambda ^{\alpha }{\bar{N}}}{K^{\alpha }} - \mu ^{\alpha } {\bar{P}} - \beta ^{\alpha }_1 {\bar{I}} - \zeta ^{\alpha }_1 {\bar{M}} &{}- \beta ^{\alpha }_1 {\bar{N}}&{}- \mu ^{\alpha } {\bar{N}}&{}- \zeta ^{\alpha }_1 {\bar{N}}&{}0\\ \\ 2 \phi _2^\alpha {\bar{N}}&{} - \phi ^{\alpha }_3 - \beta ^{\alpha }_2 {\bar{P}} + \zeta ^{\alpha }_2 {\bar{M}} - \zeta ^{\alpha }_3 {\bar{R}}&{}-\beta ^{\alpha }_2 {\bar{I}}&{}\zeta ^{\alpha }_2{\bar{I}}&{}-\zeta ^{\alpha }_3 {\bar{I}}\\ \\ \gamma {\bar{N}} &{}-\beta _3^\alpha {\bar{P}}&{}\gamma ^{\alpha } {\bar{N}} - \delta ^{\alpha } - \beta ^{\alpha }_3{\bar{I}}+\zeta ^\alpha _4{\bar{M}} &{}\zeta ^{\alpha }_4 {\bar{P}}&{}0\\ \\ \zeta ^{\alpha }_5 {\bar{M}} &{}- \zeta ^{\alpha }_6 {\bar{I}} &{}- \zeta ^{\alpha }_7 {\bar{M}}&{} \zeta ^{\alpha }_5 {\bar{N}} - \zeta ^{\alpha }_6 {\bar{I}} - \zeta ^{\alpha }_7 {\bar{P}}&{}0\\ \\ 0&{}\zeta _8^\alpha {\bar{R}} &{}0&{}0&{}\zeta ^{\alpha }_8 {\bar{I}}\\ \\ \end{pmatrix}. \end{aligned}$$

We now obtain the characteristic equation:

$$P({\bar{\lambda }})=- {\bar{\lambda }}^5+(K_1 + K_6 + K_{12} + K_{17} + K_{19}){\bar{\lambda }}^4 + (-K_1K_6 + K_2K_5 - K_1K_{12} + K_3K_{10} - K_1K_{17} + K_4K_{14} - K_6K_{12} + K_7K_{11} - K_1K_{19} - K_6K_{17} + K_8K_{15} - K_6K_{19} + K_9K_{18} - K_{12}K_{17} + K_{13}K_{16} - K_{12}K_{19} - K_{17}K_{19}){\bar{\lambda }}^3 + (K_1K_6K_{12} - K_1K_7K_{11} - K_2K_5K_{12} + K_2K_7K_{10} + K_3K_5K_{11} - K_3K_6K_{10} + K_1K_6K_{17} - K_1K_8K_{15} - K_2K_5K_{17} + K_2K_8K_{14} + K_4K_5K_{15} - K_4K_6K_{14} + K_1K_6K_{19} - K_2K_5K_{19} - K_1K_9K_{18} + K_1K_{12}K_{17} - K_1K_{13}K_{16} - K_3K_{10}K_{17} + K_3K_{13}K_{14} + K_4 K_{10}K_{16} - K_4K_{12}K_{14} + K_1K_{12}K_{19} - K_3K_{10}K_{19} + K_6K_{12}K_{17} - K_6K_{13}K_{16} - K_7K_{11}K_{17} + K_7K_{13}K_{15} + K_8K_{11}K_{16} - K_8K_{12}K_{15} + K_1K_{17}K_{19} - K_4K_{14}K_{19} + K_6K_{12}K_{19} - K_7K_{11}K_{19} - K_9K_{12}K_{18} + K_6K_{17}K_{19} - K_8K_{15}K_{19} - K_9K_{17} K_{18} + K_{12}K_{17}K_{19} - K_{13}K_{16}K_{19}){\bar{\lambda }}^2 +(-K_1K_6K_{12}K_{17} + K_1K_6K_{13}K_{16} + K_1K_7K_{11}K_{17} - K_1K_7K_{13}K_{15} - K_1K_8K_{11}K_{16} + K_1K_8K_{12}K_{15} + K_2K_5K_{12}K_{17} - K_2K_5K_{13}K_{16} - K_2K_7K_{10}K_{17}+ K_2K_7K_{13}K_{14} + K_2K_8K_{10}K_{16} - K_2K_8K_{12}K_{14} - K_3K_5K_{11}K_{17} + K_3K_5K_{13}K_{15} + K_3K_6K_{10}K_{17} - K_3K_6K_{13}K_{14} - K_3K_8K_{10}K_{15} + K_3K_8K_{11}K_{14} + K_4K_5K_{11}K_{16} - K_4K_5K_{12}K_{15} - K_4K_6K_{10}K_{16} + K_4K_6K_{12}K_{14} + K_4K_7K_{10}K_{15} - K_4K_7K_{11}K_{14} - K_1K_6K_{12}K_{19} + K_1K_7K_{11}K_{19} + K_2K_5K_{12}K_{19} - K_2K_7K_{10}K_{19} - K_3K_5K_{11}K_{19} + K_3K_6K_{10}K_{19} + K_1K_9K_{12}K_{18} - K_3K_9K_{10}K_{18} - K_1K_6K_{17}K_{19} + K_1K_8K_{15}K_{19} + K_2K_5K_{17}K_{19} - K_2K_8K_{14}K_{19} - K_4K_5K_{15}K_{19} + K_4K_6K_{14}K_{19} + K_1K_9K_{17}K_{18} - K_4K_9K_{14}K_{18} - K_1K_{12}K_{17}K_{19} + K_1K_{13}K_{16}K_{19} + K_3K_{10}K_{17}K_{19} - K_3K_{13}K_{14}K_{19} - K_{4}K_{10}K_{16}K_{19} + K_4K_{12}K_{14}K_{19} - K_6K_{12}K_{17}K_{19} + K_6K_{13}K_{16}K_{19} + K_7K_{11}K_{17}K_{19} - K_7K_{13}K_{15}K_{19} - K_8K_{11}K_{16}K_{19} + K_8K_{12}K_{15}K_{19} + K_{9}K_{12}K_{17}K_{18} - K_9K_{13}K_{16}K_{18}){\bar{\lambda }} + K_1K_6K_{12}K_{17}K_{19} - K_1K_6K_{13}K_{16}K_{19} - K_1K_7K_{11}K_{17}K_{19} + K_1K_7K_{13}K_{15}K_{19} + K_1K_8K_{11}K_{16}K_{19} - K_1K_8K_{12}K_{15}K_{19} - K_2K_5K_{12}K_{17}K_{19} + K_2K_5K_{13}K_{16}K_{19} + K_2K_7K_{10}K_{17}K_{19} - K_2K_7K_{13}K_{14}K_{19} - K_2K_8K_{10}K_{16}K_{19} + K_2K_8K_{12}K_{14}K_{19} + K_3K_5K_{11}K_{17}K_{19} - K_3K_5K_{13}K_{15}K_{19} - K_3K_6K_{10}K_{17}K_{19} + K_3K_6K_{13}K_{14}K_{19} + K_3K_8K_{10}K_{15}K_{19} - K_3K_8K_{11}K_{14}K_{19} - K_4K_5K_{11}K_{16}K_{19} + K_4K_5K_{12}K_{15}K_{19} + K_4K_6K_{10}K_{16}K_{19} - K_4K_6K_{12}K_{14}K_{19} - K_4K_7K_{10}K_{15}K_{19} + K_{4}K_7K_{11}K_{14}K_{19} - K_1K_9K_{12}K_{17}K_{18} + K_1K_9K_{13}K_{16}K_{18} + K_3K_9K_{10}K_{17}K_{18} - K_3K_9K_{13}K_{14}K_{18} - K_4K_9K_{10}K_{16}K_{18} + K_4K_9K_{12}K_{14}K_{18},$$

where, $$K_1=\lambda ^{\alpha } - \frac{2\lambda ^{\alpha }{\bar{N}}}{K^{\alpha }} - \mu ^{\alpha } {\bar{P}} - \beta ^{\alpha }_1 {\bar{I}} - \zeta ^{\alpha }_1 {\bar{M}},~K_2=- \beta ^{\alpha }_1 {\bar{N}}, ~K_3=- \mu ^{\alpha } {\bar{N}},~K_4=- \zeta ^{\alpha }_1 {\bar{N}},~K_5=2 \phi _2^\alpha {\bar{N}},$$

$$K_6=- \phi ^{\alpha }_3 - \beta ^{\alpha }_2 {\bar{P}} + \zeta ^{\alpha }_2 {\bar{M}} - \zeta ^{\alpha }_3 {\bar{R}},~K_7=-\beta ^{\alpha }_2 {\bar{I}},~K_8=\zeta ^{\alpha }_2{\bar{I}},~K_{9}=-\zeta ^{\alpha }_3 {\bar{I}}, ~K_{10}=\gamma {\bar{N}},~ K_{11}= -\beta _3^\alpha {\bar{P}},$$

$$K_{12}=\gamma ^{\alpha } {\bar{N}} - \delta ^{\alpha } - \beta ^{\alpha }_3{\bar{I}}+\zeta ^\alpha _4{\bar{M}}, ~K_{13}= \zeta ^{\alpha }_4 {\bar{P}}, ~K_{14}= \zeta ^{\alpha }_5 {\bar{M}}, ~K_{15}= - \zeta ^{\alpha }_6 {\bar{I}}, ~K_{16}=- \zeta ^{\alpha }_7 {\bar{M}},$$

$$K_{18}=K_{17}= \zeta ^{\alpha }_5 {\bar{N}} - \zeta ^{\alpha }_6 {\bar{I}} - \zeta ^{\alpha }_7 {\bar{P}},~\zeta _8^\alpha {\bar{R}},~ K_{19}= \zeta ^{\alpha }_8 {\bar{I}}.$$

This can further be reduced to:

9 $$\begin{aligned} P({\bar{\lambda }})= & {} -{\bar{\lambda }}^5+ (a_5-a_3-a_1){\bar{\lambda }}^4 +(a_3a_5+a_1a_5-a_2-a_4-a_1a_3){\bar{\lambda }}^3+(a_1a_3a_5-a_2a_3+a_2a_5-a_1a_4){\bar{\lambda }}^2\nonumber \\{} & {} +(a_2a_3a_5+a_1a_4a_5-a_2a_4){\bar{\lambda }}+a_2a_4a_5, \end{aligned}$$

where,

$$a_1=-K_1,~~a_2=-K_2K_5+K_2K_7K_{10}+K_3K_7K_{11}-K_3K_{10}+K_3K_{13}K_{14}+K_4K_{10}K_{16}-K_4K_{12}K_{14}-K_{12}K_{17}-K_{13}K_{16}-K_7K_{11}K_{17}+K_7K_{13}K_{15}+K_8K_{11}K_{16}-K_8K_{15}-K_4K_{14}+K_{12}K_{19}-K_7K_{11}K_{19}-K_9K_{18}+K_{17}K_{19}-K_8K_{15}-K_{13}K_{16},~~a_3=-K_6,~~a_4=-K_7K{11}+K_{12}K{17}-K_{13}K{16}+K_{12}K{19}+K_{17}K{19},~~ a_5=K_{12}+K_{17}+K_{19}.$$

#### Theorem 4.1

The equilibrium point $E_{14}$ is stable if $a_1>0, a_2>0, a_3>0, a_4>0$ and $a_5<0$.

#### Proof

We can further simplify (9) and arrive at:

$$\begin{aligned} P({\bar{\lambda }})=({\bar{\lambda }}^2+a_1 {\bar{\lambda }}+a_2)({\bar{\lambda }}^2+a_3{\bar{\lambda }}+a_4)(a_5-{\bar{\lambda }}). \end{aligned}$$

The eigenvalue ${\bar{\lambda }}=a_5$ will be negative if $a_5<0$. If $a_1>0,~a_2>0,~a_3>0,~a_4>0,$ then according to the Routh-Hurwitz criterion, the other eigenvalues have negative real part. Therefore, the equilibrium point $E_{14}$ is stable. $\square$

## Optimization

### Fractional-order lung cancer model with drug interventions

In this section, we propose an optimization model (10) that aims to determine optimal drug dosages for the combination therapy. This involves carefully adjusting the dosages of immunotherapy and targeted agents, such as those targeting antiangiogenesis, EGFR mutations, and ALK translocations. The objective is to strike a balance between maximizing treatment efficacy and minimizing potential side effects, ultimately enhancing the therapeutic outcomes in the context of heterogeneous lung cancer progression. The optimization problem is given by:

Minimize:

$$\begin{aligned} \quad J = \int _{0}^{T_f} \left[ a_1^{\alpha } N(t) + a_2^{\alpha } I(t) + a_3^{\alpha } P(t) + a_4^{\alpha } M(t) + a_5^{\alpha } R(t) + b_1^{\alpha } D_I^2(t) + b_2^{\alpha } D_T^2(t) \right] dt, \end{aligned}$$

subject to:

10 $$\begin{aligned} _{0}^{c}D_{t}^{\alpha }N(t)&= \lambda ^{\alpha }N(t)\left( 1 - \frac{N(t)}{K^{\alpha }}\right) - \mu ^{\alpha } N(t)P(t) - \beta ^{\alpha }_1N(t)I(t) - \zeta ^{\alpha }_1 N(t)M(t) - \chi ^{\alpha }_1 N(t)D_I(t), \\ _{0}^{c}D_{t}^{\alpha }I(t)&= \phi ^{\alpha }_1I_0 + \phi ^{\alpha }_2N(t)^2 - \phi ^{\alpha }_3I(t) - \beta ^{\alpha }_2I(t)P(t) + \zeta ^{\alpha }_2 I(t)M(t) - \zeta ^{\alpha }_3 I(t)R(t) + \chi ^{\alpha }_2 I(t)D_T(t), \\ _{0}^{c}D_{t}^{\alpha }P(t)&= \gamma ^{\alpha } N(t)P(t) - \delta ^{\alpha } P(t) - \beta ^{\alpha }_3I(t)P(t) + \zeta ^{\alpha }_4 P(t)M(t) - \chi ^{\alpha }_3 P(t)D_T(t), \\ _{0}^{c}D_{t}^{\alpha }M(t)&= \zeta ^{\alpha }_5 N(t)M(t) - \zeta ^{\alpha }_6 I(t)M(t) - \zeta ^{\alpha }_7 P(t)M(t) + \chi ^{\alpha }_4 M(t)D_T(t) + \chi ^{\alpha }_5 M(t)D_I(t), \\ _{0}^{c}D_{t}^{\alpha }R(t)&= \zeta ^{\alpha }_8 I(t)R(t) + \chi ^{\alpha }_6 R(t)D_I(t), \end{aligned}$$

where $T_f$ is the total treatment time, $D_I$ represents the drug dosage of the immunotherapy treatment and $D_T$ represents the drug dosage of the targeted treatment, with non-negativity constraints on all variables. The coefficients $\chi _1^\alpha , \chi _2^\alpha , \chi _3^\alpha , \chi _4^\alpha , \chi _5^\alpha , \chi _6^\alpha$ represent the strength of the respective drug interventions. The weights $a^{\alpha }_1, a^{\alpha }_2, a^{\alpha }_3, a^{\alpha }_4, a^{\alpha }_5$ and $b^{\alpha }_1, b^{\alpha }_2$ in the objective function determine the importance of minimizing each state variable and controlling drug dosages, respectively. Adjusting these coefficients and weights allows for customization based on clinical goals and trial results. We can define the Hamiltonian function for system (10) as:

11 $$\begin{aligned} {\mathcal {H}}(t)&=a_1^{\alpha } N(t)+a_2^{\alpha } I(t)+a_3^{\alpha } P(t) +a_4^{\alpha } M(t)+a_5^{\alpha } R(t)+b_1^{\alpha } D_I^2(t)+b_2^{\alpha } D_T^2(t)\\&\quad +\lambda _1\bigg ( \lambda ^{\alpha }N(t)\left( 1 - \frac{N(t)}{K^{\alpha }}\right) - \mu ^{\alpha } N(t)P(t) - \beta ^{\alpha }_1N(t)I(t) - \zeta ^{\alpha }_1 N(t)M(t) - \chi ^{\alpha }_1 N(t)D_I(t) \bigg ) \\&\quad +\lambda _2\bigg (\phi ^{\alpha }_1I_0 + \phi ^{\alpha }_2N(t)^2 - \phi ^{\alpha }_3I(t) - \beta ^{\alpha }_2I(t)P(t) + \zeta ^{\alpha }_2 I(t)M(t) - \zeta ^{\alpha }_3 I(t)R(t) + \chi ^{\alpha }_2 I(t)D_T(t) \bigg )\\&\quad +\lambda _3\bigg (\gamma ^{\alpha } N(t)P(t) - \delta ^{\alpha } P(t) - \beta ^{\alpha }_3I(t)P(t) + \zeta ^{\alpha }_4 P(t)M(t) - \chi ^{\alpha }_3 P(t)D_T(t) \bigg ) \\&\quad +\lambda _4\bigg (\zeta ^{\alpha }_5 N(t)M(t) - \zeta ^{\alpha }_6 I(t)M(t) - \zeta ^{\alpha }_7 P(t)M(t) + \chi ^{\alpha }_4 M(t)D_T(t) + \chi ^{\alpha }_5 M(t)D_I(t) \bigg )\\&\quad +\lambda _5\bigg (\zeta ^{\alpha }_8 I(t)R(t) + \chi ^{\alpha }_6 R(t)D_I(t)\bigg ), \end{aligned}$$

where $\lambda _i(t), i=1, \ldots , 5$ are adjoint variables and satisfy the following equations using Pontryagin’s maximum principle^34,43^:

$$\begin{aligned} \frac{d \lambda _1(t)}{dt}&=-\frac{\partial {\mathcal {H}}(t)}{\partial N(t)}=-\bigg \{a_1^{\alpha }+\lambda _1 \bigg [\lambda ^{\alpha }-\frac{2\lambda ^{\alpha }}{K^{\alpha }}-\mu ^{\alpha }P(t)-\beta _1^{\alpha } I(t)-\zeta _1^{\alpha }M(t)-\chi _1^{\alpha }D_I(t)\bigg ]\\&\quad +\lambda _2 \bigg [2 \phi _2^{\alpha } N(t)\bigg ]+\lambda _3 \bigg [\gamma ^{\alpha } P(t)\bigg ]+\lambda _4 \bigg [\zeta _5^{\alpha } M(t)\bigg ]\bigg \},\\ \frac{d \lambda _2(t)}{dt}&=-\frac{\partial {\mathcal {H}}(t)}{\partial I(t)}=-\bigg \{ a_2^{\alpha }+\lambda _1\bigg [-\beta _1^{\alpha } N(t)\bigg ]+\lambda _2\bigg [-\phi _3^{\alpha }-\beta _2^{\alpha }P(t)+\zeta _2^{\alpha }M(t)-\zeta _3^{\alpha }R(t)+\chi _2^{\alpha } D_T(t)\bigg ]\\&\quad +\lambda _4\bigg [-\zeta _6 ^{\alpha }M(t)\bigg ]+\lambda _5\bigg [\zeta _8^{\alpha }R(t)\bigg ] \bigg \},\\ \frac{d \lambda _3(t)}{dt}&=-\frac{\partial {\mathcal {H}}(t)}{\partial P(t)}=-\bigg \{a_3^{\alpha }+\lambda _1\bigg [-\mu ^{\alpha }N(t)\bigg ]+\lambda _2\bigg [-\beta _2^{\alpha }I(t)\bigg ]+\lambda _3\bigg [\gamma ^{\alpha }N(t)-\delta ^{\alpha }-\beta _3^{\alpha } I(t)+\zeta _4^{\alpha } M(t)\\&\quad -\chi _3^{\alpha }D_T(t)\bigg ]+\lambda _4 \bigg [-\zeta _7^{\alpha } M(t)\bigg ]\bigg \},\\ \frac{d \lambda _4(t)}{dt}&=-\frac{\partial {\mathcal {H}}(t)}{\partial M(t)}=-\bigg \{a_4^{\alpha }+\lambda _1\bigg [-\zeta _1^{\alpha } N(t)\bigg ]+\lambda _2\bigg [\zeta _2^{\alpha I(t)}\bigg ]+\lambda _3 \bigg [\zeta _4^{\alpha } P(t)\bigg ]+\lambda _4 \bigg [\zeta _5^{\alpha }N(t)-\zeta _6^{\alpha } I(t)-\zeta _7^{\alpha }P(t)\\&\quad +\chi _4^{\alpha }D_T(t)+\chi _5^{\alpha } D_T(t)\bigg ]\bigg \},\\ \frac{d \lambda _5(t)}{dt}&=-\frac{\partial {\mathcal {H}}(t)}{\partial R(t)}=-\bigg \{a_5^{\alpha }+\lambda _2\bigg [-\zeta _3^{\alpha } I(t)\bigg ]+\lambda _5\bigg [\zeta _8^{\alpha } I(t)+\chi _6^{\alpha }D_I(t)\bigg ]\bigg \}, \end{aligned}$$

and the transversality conditions are $\lambda _i(T_f)=0, i=1, \ldots , 5$.Assume that $D_I^*$ and $D_T^*$ are optimal values of control variables.The optimal control functions are derived as follows:

12 $$\begin{aligned} \frac{\partial {\mathcal {H}}(t)}{\partial D_I(t)}=0 \quad \Rightarrow \quad 2 b_1^{\alpha } D_I(t) -\chi _1^{\alpha } \lambda _1(t) N(t)+\chi _5^{\alpha }\lambda _4(t) M(t) +\chi _6^{\alpha }\lambda _5(t) R(t)=0. \end{aligned}$$

So, we get:

$$\begin{aligned} D_I(t)= \frac{\chi _1^{\alpha } \lambda _1(t) N(t)-\chi _5^{\alpha }\lambda _4(t) M(t)-\chi _6^{\alpha }\lambda _5(t) R(t)}{2 b_1^{\alpha }}=\Delta _I(t). \end{aligned}$$

Similarly, we can get:

13 $$\begin{aligned} \frac{\partial {\mathcal {H}}(t)}{\partial D_T(t)}=0 \quad \Rightarrow \quad 2 b_2^{\alpha } D_T(t) +\chi _2^{\alpha } \lambda _2(t) I(t)-\chi _3^{\alpha }\lambda _3(t) P(t)+\chi _4^{\alpha } \lambda _4(t) M(t)=0. \end{aligned}$$

Thus, we get:

$$\begin{aligned} D_T(t)=- \frac{\chi _2^{\alpha } \lambda _2(t) I(t)-\chi _3^{\alpha }\lambda _3(t) P(t)+\chi _4^{\alpha } \lambda _4(t) M(t)}{2 b_2^{\alpha }}=\Delta _T(t). \end{aligned}$$

Therefore, we have:

$$\begin{aligned} D_I^*= \left\{ \begin{array}{ll} 0, &{} \text {if} \,\, \Delta _I \le 0, \\ \Delta _I, &{} \text {if} \,\, 0< \Delta _I< 0, \\ 1, &{} \text {if} \,\, 1\le \Delta _I, \end{array} \right. \quad D_T^*= \left\{ \begin{array}{ll} 0, &{} \text {if} \,\, \Delta _T \le 0, \\ \Delta _T, &{} \text {if} \,\, 0< \Delta _T < 0, \\ 1, &{} \text {if} \,\, 1\le \Delta _T. \end{array} \right. \end{aligned}$$

In a new notation, we have:

$$\begin{aligned} D_I^*=\min \{\max \{0, \Delta _I\}, 1\}, \quad D_T^*=\min \{\max \{0, \Delta _T\}, 1\}. \end{aligned}$$

The second-order derivatives of Eqs. (12) and (13) are:

$$\begin{aligned} \frac{\partial ^2 {\mathcal {H}}(t)}{\partial D_I^2}=2 b_1^{\alpha }>0, \quad \frac{\partial ^2 {\mathcal {H}}(t)}{\partial D_T^2}=2 b_2^{\alpha }>0. \end{aligned}$$

This implies that the optimal problem is minimized at $D_I$ and $D_T$. Finally, we have the following optimal problem:

14 $$\begin{aligned} _{0}^{c}D_{t}^{\alpha }N(t)&= \lambda ^{\alpha }N(t)\left( 1 - \frac{N(t)}{K^{\alpha }}\right) - \mu ^{\alpha } N(t)P(t) - \beta ^{\alpha }_1N(t)I(t) - \zeta ^{\alpha }_1 N(t)M(t) - \chi ^{\alpha }_1 N(t)D_I^*(t), \nonumber \\ _{0}^{c}D_{t}^{\alpha }I(t)&= \phi ^{\alpha }_1I_0 + \phi 
^{\alpha }_2N^2(t) - \phi ^{\alpha }_3I(t) - \beta ^{\alpha }_2I(t)P(t) + \zeta ^{\alpha }_2 I(t)M(t) - \zeta ^{\alpha }_3 I(t)R(t) + \chi ^{\alpha }_2 I(t)D_T^*(t), \nonumber \\ _{0}^{c}D_{t}^{\alpha }P(t)&= \gamma ^{\alpha } N(t)P(t) - \delta ^{\alpha } P(t) - \beta ^{\alpha }_3I(t)P(t) + \zeta ^{\alpha }_4 P(t)M(t) - \chi ^{\alpha }_3 P(t)D_T^*(t), \nonumber \\ _{0}^{c}D_{t}^{\alpha }M(t)&= \zeta ^{\alpha }_5 N(t)M(t) - \zeta ^{\alpha }_6 I(t)M(t) - \zeta ^{\alpha }_7 P(t)M(t) + \chi ^{\alpha }_4 M(t)D_T^*(t) + \chi ^{\alpha }_5 M(t)D_I^*(t), \nonumber \\ _{0}^{c}D_{t}^{\alpha }R(t)&= \zeta ^{\alpha }_8 I(t)R(t) + \chi ^{\alpha }_6 R(t)D_I^*(t), \end{aligned}$$

15 $$\begin{aligned} \frac{d \lambda _1(t)}{dt}&=-a_1^{\alpha }-\lambda _1(t) (\lambda ^{\alpha }-\frac{2\lambda ^{\alpha }}{K^{\alpha }}-\mu ^{\alpha }P(t)-\beta _1^{\alpha } I(t)-\zeta _1^{\alpha }M(t)-\chi _1^{\alpha }D_I(t)) -2 \phi _2^{\alpha }\lambda _2 (t) N(t)\nonumber \\&\quad - \gamma ^{\alpha } \lambda _3(t) P(t)- \zeta _5^{\alpha } \lambda _4(t) M(t),\nonumber \\ \frac{d \lambda _2(t)}{dt}&=- a_2^{\alpha }+\beta _1^{\alpha } \lambda _1(t) N(t)-\lambda _2(t)(-\phi _3^{\alpha }-\beta _2^{\alpha }P(t)+\zeta _2^{\alpha }M(t)-\zeta _3^{\alpha }R(t)+\chi _2^{\alpha } D_T(t))\nonumber \\&\quad +\zeta _6 ^{\alpha } \lambda _4(t) M(t)+\zeta _8^{\alpha } \lambda _5(t) R(t),\nonumber \\ \frac{d \lambda _3(t)}{dt}&=-a_3^{\alpha }+ \mu ^{\alpha } \lambda _1(t) N(t)+\beta _2^{\alpha } \lambda _2(t) I(t)-\lambda _3(t)(\gamma ^{\alpha }N(t)-\delta ^{\alpha }-\beta _3^{\alpha } I(t)+\zeta _4^{\alpha } M(t)\nonumber \\&\quad -\chi _3^{\alpha }D_T(t))+ \zeta _7^{\alpha } \lambda _4(t) M(t),\nonumber \\ \frac{d \lambda _4(t)}{dt}&=-a_4^{\alpha }+\zeta _1^{\alpha } \lambda _1(t) N(t)-\zeta _2^{\alpha }\lambda _2(t) I(t)-\zeta _4^{\alpha } \lambda _3(t) P(t)+\lambda _4(t) (\zeta _5^{\alpha }N(t)-\zeta _6^{\alpha } I(t)-\zeta _7^{\alpha }P(t)\nonumber \\&\quad +\chi _4^{\alpha }D_T(t)+\chi _5^{\alpha } D_T(t)),\nonumber \\ \frac{d \lambda _5(t)}{dt}&=-a_5^{\alpha } +\zeta _3^{\alpha } \lambda _2(t) I(t)-\lambda _5(t)(\zeta _8^{\alpha } I(t)+\chi _6^{\alpha }D_I(t)), \end{aligned}$$

16 $$\begin{aligned} D_I^*&=\min \{\max \{0, \Delta _I\}, 1\}, \quad D_T^*=\min \{\max \{0, \Delta _T\}, 1\}, \end{aligned}$$

subject to the conditions:

17 $$\begin{aligned} &N(0)=N_0, \quad I(0)=I_0, \quad P(0)=P_0, \quad M(0)=M_0, \quad R(0)=R_0, \\&\lambda _i(T_f)=0, \quad i=1, \ldots , 5. \end{aligned}$$

Problem (14)–(17) can be solved using an efficient numerical algorithm.

## Feedback control with PID controller

In this section, we introduce a feedback control mechanism employing a Proportional-Integral-Derivative (PID) controller for the combination therapy proposed in the optimization model (10). The PID controller aims to regulate the drug dosages dynamically, allowing the system to adapt to the evolving characteristics of lung cancer progression. The PID controller manipulates the drug dosages $D_I(t)$ and $D_T(t)$ based on the error signal, which is the difference between the desired state and the actual state of the system. The PID controller manipulates the drug dosages based on the error signals, which are the differences between the desired and actual states. The control signal is computed as follows:

$$\begin{aligned} u_I(t)&= K_p e_I(t) + K_i \int _{0}^{t} e_I(\tau ) d\tau + K_d \frac{de_I(t)}{dt}, \\ u_T(t)&= K_p e_T(t) + K_i \int _{0}^{t} e_T(\tau ) d\tau + K_d \frac{de_T(t)}{dt}, \end{aligned}$$

where

- $K_p$, $K_i$, and $K_d$ are the proportional, integral, and derivative gains, respectively.
- $e_I(t)$ and $e_T(t)$ are the error signals for immunotherapy and targeted therapy, respectively.

The control signal is then used to adjust the drug dosages as follows:

$$\begin{aligned} D_I(t)&= D_I(t) + u_I(t), \\ D_T(t)&= D_T(t) + u_T(t). \end{aligned}$$

The objective is to minimize the cost function $J$ over the treatment period $T_f$, accounting for the PID control terms:

$$\begin{aligned} J = \int _{0}^{T_f} \left[ a^{\alpha }_1N(t) + a^{\alpha }_2I(t) + a^{\alpha }_3P(t) + a^{\alpha }_4M(t) + a^{\alpha }_5R(t) + b^{\alpha }_1D_I^2(t) + b^{\alpha }_2D_T^2(t) + c^{\alpha }_1u_I^2(t) + c^{\alpha }_2u_T^2(t) \right] dt, \end{aligned}$$

subject to:

18 $$\begin{aligned} _{0}^{c}D_{t}^{\alpha }N(t)&= \lambda ^{\alpha }N(t)\left( 1 - \frac{N(t)}{K^{\alpha }}\right) - \mu ^{\alpha } N(t)P(t) - \beta ^{\alpha }_1N(t)I(t) - \zeta ^{\alpha }_1 N(t)M(t) - \chi ^{\alpha }_1 N(t)\left( D_I(t) + u_I(t)\right) , \\ _{0}^{c}D_{t}^{\alpha }I(t)&= \phi ^{\alpha }_1I_0 + \phi ^{\alpha }_2N^2(t) - \phi ^{\alpha }_3I(t) - \beta ^{\alpha }_2I(t)P(t) + \zeta ^{\alpha }_2 I(t)M(t) - \zeta ^{\alpha }_3 I(t)R(t) + \chi ^{\alpha }_2 I(t)\left( D_T(t) + u_T(t)\right) , \\ _{0}^{c}D_{t}^{\alpha }P(t)&= \gamma ^{\alpha } N(t)P(t) - \delta ^{\alpha } P(t) - \beta ^{\alpha }_3I(t)P(t) + \zeta ^{\alpha }_4 P(t)M(t) - \chi ^{\alpha }_3 P(t)\left( D_T(t) + u_T(t)\right) , \\ _{0}^{c}D_{t}^{\alpha }M(t)&= \zeta ^{\alpha }_5 N(t)M(t) - \zeta ^{\alpha }_6 I(t)M(t) - \zeta ^{\alpha }_7 P(t)M(t) + \chi ^{\alpha }_4 M(t)(D_T(t) + u_T(t)) + \chi ^{\alpha }_5 M(t)\left( D_I(t) + u_I(t)\right) , \\ _{0}^{c}D_{t}^{\alpha }R(t)&= \zeta ^{\alpha }_8 I(t)R(t) + \chi ^{\alpha }_6 R(t)\left( D_I(t) + u_I(t)\right) , \end{aligned}$$

where $u_{I}(t)$ and $u_{T}(t)$ are the control signals from the PID controller associated with immunotherapy and targeted therapy, respectively.

To integrate the PID controller with the optimization model in (10), the updated drug dosages ($D_I(t)$ and $D_T(t)$) are fed back into the model’s dynamics. The combination of the optimization model and the PID controller allows for a dynamic and adaptive approach to drug dosage adjustments, enhancing the therapeutic outcomes while considering the evolving nature of lung cancer progression.The updated drug dosages and control signals are fed back into the fractional-order lung cancer model, creating a closed-loop system. This allows for dynamic adjustments of drug dosages in response to the system’s behavior, resulting in a more adaptive and responsive treatment strategy. Adjustments to the PID gains ($K_p$, $K_i$, $K_d$) can be made based on clinical feedback and the specific requirements of the combination therapy. The incorporation of a PID controller provides a feedback mechanism that enhances the adaptability of the combination therapy, ensuring a more responsive and effective treatment strategy in the face of heterogeneous lung cancer progression. The integration of PID feedback time control into the optimization model enhances the adaptability of the combination therapy, providing a mechanism to dynamically regulate drug dosages in real-time, ultimately improving therapeutic outcomes.

## Patient stratification and personalized medicine

Incorporating mathematical formulations enhances the precision and clarity of patient stratification within the proposed model:

### Patient characteristics and stratification

The fractional-order lung cancer model accounts for patient-specific parameters, denoted as $\varvec{\theta }$, including tumor growth rates ($\lambda ^{\alpha }$), mutation rates ($\beta ^{\alpha }_1, \beta ^{\alpha }_2, \beta ^{\alpha }_3$), and intervention strengths ($\chi _1^\alpha , \chi _2^\alpha , \chi _3^\alpha , \chi _4^\alpha , \chi _5^\alpha , \chi _6^\alpha$). Patient stratification involves identifying optimal parameter sets $\varvec{\theta }_i$ for different patient subpopulations based on characteristics such as genetic profiles and initial conditions.

$$\begin{aligned} \varvec{\theta }_i = \arg \min _{\varvec{\theta }} J_i. \end{aligned}$$

Here, $J_i$ represents the cost function specific to the $i$-th patient subgroup, emphasizing the importance of minimizing treatment costs while achieving therapeutic goals.

### Adaptive treatment protocols

The PID control strategy dynamically adjusts drug dosages based on error signals $e_I(t)$ and $e_T(t)$ associated with immunotherapy and targeted therapy, respectively. The adaptive control law is expressed as:

$$\begin{aligned} u_I(t)= & {} K_p e_I(t) + K_i \int _{0}^{t} e_I(\tau ) d\tau + K_d \frac{de_I(t)}{dt},\\ u_T(t)= & {} K_p e_T(t) + K_i \int _{0}^{t} e_T(\tau ) d\tau + K_d \frac{de_T(t)}{dt}. \end{aligned}$$

The PID controller continuously optimizes drug dosages $D_I(t)$ and $D_T(t)$ in response to changing patient conditions, ensuring adaptability and personalized treatment.

### Future directions in personalized medicine

Future enhancements may involve incorporating real-time patient data, denoted as ${\varvec{X}}(t)$, into the model:

$$\begin{aligned} {\varvec{X}}(t) = [X_1(t), X_2(t), \ldots , X_n(t)]. \end{aligned}$$

Where $X_i(t)$ represents additional patient-specific variables or biomarkers. The evolution of ${\varvec{X}}(t)$ can be modeled to capture emerging information, enabling real-time adaptation of the therapeutic strategy.

## Cost-benefit analysis

A mathematical framework for cost-benefit analysis involves quantifying direct and indirect costs within the context of the fractional-order lung cancer model:

### Direct treatment costs

Direct costs ($C_{\text {direct}}$) are computed as the sum of drug costs, monitoring expenses, and other medical services:

19 $$\begin{aligned} C_{\text {direct}} = \int _{0}^{T_f} (b^{\alpha }_1D_I(t) + b^{\alpha }_2D_T(t) + c^{\alpha }_1u_I(t) + c^{\alpha }_2u_T(t)) \, dt. \end{aligned}$$

The optimization objective involves minimizing $C_{\text {direct}}$ while maintaining therapeutic efficacy, represented by the integral of the treatment-related variables over the treatment period.

### Indirect costs and quality of life

Indirect costs ($C_{\text {indirect}}$) encompass factors influencing societal well-being. Quality-adjusted life years (QALY) can be introduced to assess improvements in patient quality of life ($QoL(t)$):

20 $$\begin{aligned} C_{\text {indirect}} = \int _{0}^{T_f} QoL(t) \, dt. \end{aligned}$$

The cost-benefit ratio is then expressed as the ratio of the total benefits to the total costs:

21 $$\begin{aligned} \text {Cost-Benefit Ratio} = \frac{C_{\text {indirect}}}{C_{\text {direct}} + C_{\text {indirect}}}. \end{aligned}$$

### Comparative analysis

A comparative analysis involves evaluating the cost-benefit ratio for the proposed therapy ($\text {Cost-Benefit Ratio}_{\text {proposed}}$) against existing treatments ($\text {Cost-Benefit Ratio}_{\text {existing}}$). The ratio comparison guides decision-makers in assessing the economic feasibility of the proposed therapy.

## Long-term effects and survivorship

Mathematical considerations for long-term effects and survivorship involve extending the model dynamics and control strategy over extended time frames:

### Treatment-related long-term effects

The model’s long-term effects ($E(t)$) are incorporated as additional state variables capturing cumulative treatment-related impacts. The differential equation governing the evolution of long-term effects ($E(t)$) is given by:

22 $$\begin{aligned} \frac{dE(t)}{dt} = -\theta _1 E(t) + \theta _2 D_I(t) + \theta _3 D_T(t). \end{aligned}$$

The PID controller adapts drug dosages to minimize $E(t)$, reflecting a dynamic approach to mitigating cumulative toxicities. In the Treatment-Related Long-Term Effects section, the parameters $\theta _1$, $\theta _2$, and $\theta _3$ are used to model the dynamics of the long-term effects ($E(t)$) in the fractional-order lung cancer model. $\theta _1$ represents the decay or reduction rate of the long-term effects. A higher value of $\theta _1$ implies a faster decay, indicating a more rapid resolution or reduction of treatment-related impacts over time. $\theta _2$ represents the contribution of the immunotherapy dosage $D_I(t)$ to the accumulation of long-term effects. This parameter captures the extent to which immunotherapy contributes to the persistent effects experienced by the patient. $\theta _3$ represents the contribution of the targeted therapy dosage $D_T(t)$ to the accumulation of long-term effects. Similar to $\theta _2$, this parameter quantifies the impact of targeted therapy on the persistence of long-term effects.

This equation reflects a balance between the decay of long-term effects ($-\theta _1 E(t)$) and the contributions from immunotherapy ($\theta _2 D_I(t)$) and targeted therapy ($\theta _3 D_T(t)$) to the accumulation of these effects over time. Adjusting the values of $\theta _1$, $\theta _2$, and $\theta _3$ allows for modeling different schemes and treatment strategies with varying impacts on long-term outcomes.

### Survivorship and quality of life

Survivorship considerations involve assessing the impact on overall quality of life ($QoL(t)$) throughout the extended survivorship period:

23 $$\begin{aligned} \frac{dQoL(t)}{dt} = \delta _0 E(t) - \eta QoL(t). \end{aligned}$$

Here, $QoL(t)$ accounts for factors such as functional status and mental health, providing a holistic representation of survivorship outcomes.

### Post-treatment monitoring and adaptive strategies

Post-treatment monitoring involves extending the PID control strategy beyond the treatment period ($T_f$):

$$\begin{aligned} {\varvec{u}}(t) = {\left\{ \begin{array}{ll} \text {PID Control Law}, &{} \text {if } t \le T_f, \\ 0, &{} \text {if } t > T_f. \end{array}\right. } \end{aligned}$$

This ensures that adaptive strategies continue to be employed during survivorship, addressing potential late-onset complications and supporting sustained positive outcomes.

## Numerical analysis

To numerically solve systems (1), (2), and (10), we consider the initial value problem in (1):

$$\begin{aligned} {_0^c}D_t^{\alpha }{\textbf{y}}(t)={\textbf{f}}(t, {\textbf{y}}(t)), \quad {\textbf{y}}(0)={\textbf{y}}_0. \end{aligned}$$

Employing the Riemann–Liouville integral operator in Definition 2.2, we get that:

24 $$\begin{aligned} {\textbf{y}}(t)-{\textbf{y}}_0=\frac{1}{\Gamma (\alpha )}\int _0^t (t-s)^{\alpha -1}\, {\textbf{f}}(s, {\textbf{y}}(s))\, ds. \end{aligned}$$

After substituting $t=t_{n+1}$ into Eq. (24) and subtracting two obtained equations, we can write:

25 $$\begin{aligned} {\textbf{y}}(t_{n+1})={\textbf{y}}_0+\frac{1}{\Gamma (\alpha )} \sum \limits _{m=0}^n \int _{t_m}^{t_{m+1}} (t_{n+1}-s)^{\alpha -1}\, {\textbf{f}}(s, {\textbf{y}}(s))\, ds, \end{aligned}$$

where $t_j=jh, j=0, 1, \ldots , N$ and $h=T_f/N$ is the step size. We now approximate the function ${\textbf{f}}(s, {\textbf{y}}(s))$ on the interval $[t_m, t_{m+1}]$ using the two-step Lagrange polynomial interpolation:

26 $$\begin{aligned} {\textbf{f}}(s, {\textbf{y}}(s))&\approx \frac{s-t_{m+1}}{t_m-t_{m+1}}{\textbf{f}}(t_m, {\textbf{y}}_m)+ \frac{s-t_{m}}{t_{m+1}-t_{m}}{\textbf{f}}(t_{m+1}, {\textbf{y}}_{m+1})\\&=-\frac{s-t_{m+1}}{h}{\textbf{f}}(t_m, {\textbf{y}}_m)+ \frac{s-t_{m}}{h}{\textbf{f}}(t_{m+1}, {\textbf{y}}_{m+1}), \end{aligned}$$

where ${\textbf{y}}_{k}={\textbf{y}}(t_k)$. Using (25) and (26), we have

27 $$\begin{aligned} {\textbf{y}}_{n+1}&={\textbf{y}}_0 + \frac{1}{h\, \Gamma (\alpha )} \bigg \{ \sum \limits _{m=0}^{n} \int _{t_m}^{t_{m+1}} (t_{n+1}-s)^{\alpha -1}\, (s-t_m)\, {\textbf{f}}(t_{m+1}, {\textbf{y}}_{m+1})\, ds \\&\quad -\sum \limits _{m=0}^{n} \int _{t_m}^{t_{m+1}} (t_{n+1}-s)^{\alpha -1}\, (s-t_{m+1})\, {\textbf{f}}(t_{m}, {\textbf{y}}_{m})\, ds \bigg \}, \quad n=0, 1, \ldots , N.\end{aligned}$$

Using integration by parts, (27) is converted into the following formula:

28 $$\begin{aligned} {\textbf{y}}_{n+1}&={\textbf{y}}_0 + \frac{h^{\alpha }}{\Gamma (\alpha +2)} \sum \limits _{m=0}^{n} \bigg \{ [(n-m+1)^{\alpha +1}-(n-m)^{\alpha }(n-m+\alpha +1)]\, {\textbf{f}}(t_{m+1}, {\textbf{y}}_{m+1}^p) \\&\quad -[(n-m+1)^{\alpha }(n-m-\alpha )-(n-m)^{\alpha +1}]\, {\textbf{f}}(t_{m}, {\textbf{y}}_{m})\bigg \}, \quad n=0, 1, \ldots , N. \end{aligned}$$

Due to appearing ${\textbf{y}}_{m+1}$ in the right side of (28), this formula is an implicit formula and values of ${\textbf{y}}_{m+1}$ should be predicted (as ${\textbf{y}}_{m+1}^p$). Thus, formula (28) will be a corrector formula. In formula (25), we use the rectangle rule for the integral part and obtain the following predictor formula:

29 $$\begin{aligned} {\textbf{y}}_{n+1}^p ={\textbf{y}}_{0}+\frac{h^{\alpha }}{\Gamma (\alpha +1)} \sum \limits _{m=0}^{n} B_{\frac{n(n+1)}{2}+m+1}\,{\textbf{f}}(t_{m}, {\textbf{y}}_{m}), \quad n=0, 1, \ldots , N, \end{aligned}$$

where,

$$\begin{aligned} B_{\frac{n(n+1)}{2}+m+1}=(n-m+1)^{\alpha }-(n-m)^{\alpha }, \quad n=0, 1, \ldots , N, \,\, m=0, 1, \ldots , n. \end{aligned}$$

Therefore, the numerical formula for system (1) is as follows:

**The predictor formula:**

$$\begin{aligned} N_{n+1}^p&=N_0+\frac{h^{\alpha }}{\Gamma (\alpha +1)} \sum \limits _{m=0}^n B_{\frac{n(n+1)}{2}+m+1}\,\bigg \{ \lambda ^{\alpha }N_m\left( 1 - \frac{N_m}{K^{\alpha }}\right) - \mu ^{\alpha } N_m P_m - \beta ^{\alpha }_1N_m I_m - \zeta ^{\alpha }_1 N_m M_m\bigg \}, \\ I_{n+1}^p&=I_0+ \frac{h^{\alpha }}{\Gamma (\alpha +1)} \sum \limits _{m=0}^n B_{\frac{n(n+1)}{2}+m+1}\,\bigg \{ \phi ^{\alpha }_1I_0 + \phi ^{\alpha }_2N_m^2 - \phi ^{\alpha }_3I_m - \beta ^{\alpha }_2I_m P_m + \zeta ^{\alpha }_2 I_m M_m - \zeta ^{\alpha }_3 I_m R_m\bigg \},\\ P_{n+1}^p&=P_0+ \frac{h^{\alpha }}{\Gamma (\alpha +1)} \sum \limits _{m=0}^n B_{\frac{n(n+1)}{2}+m+1}\,\bigg \{ \gamma ^{\alpha } N_m P_m - \delta ^{\alpha } P_m - \beta ^{\alpha }_3I_m P_m + \zeta ^{\alpha }_4 P_m M_m\bigg \},\\ M_{n+1}^p&=M_0 +\frac{h^{\alpha }}{\Gamma (\alpha +1)} \sum \limits _{m=0}^n B_{\frac{n(n+1)}{2}+m+1}\,\bigg \{\zeta ^{\alpha }_5 N_m M_m - \zeta ^{\alpha }_6 I_m M_m - \zeta ^{\alpha }_7 P_m M_m\bigg \},\\ R_{n+1}^p&=R_0+\frac{h^{\alpha }}{\Gamma (\alpha +1)} \sum \limits _{m=0}^n B_{\frac{n(n+1)}{2}+m+1}\,\bigg \{\zeta ^{\alpha }_8 I_m R_m \bigg \}. \end{aligned}$$

**The corrector formula:**

$$\begin{aligned} N_{n+1}&=N_0+\frac{h^{\alpha }}{\Gamma (\alpha +2)} \sum \limits _{m=0}^n \bigg [ ((n-m+1)^{\alpha +1}-(n-m)^{\alpha }(n-m+\alpha +1))\bigg \{ \lambda ^{\alpha }N^p_{m+1}\left( 1 - \frac{N^p_{m+1}}{K^{\alpha }}\right) \\&\quad - \mu ^{\alpha } N_{m+1}P_{m+1} ^p- \beta ^{\alpha }_1N_{m+1}^p I_{m+1}^p - \zeta ^{\alpha }_1 N_{m+1}^p M_{m+1}^p \bigg \}\\&\quad -((n-m+1)^{\alpha }(n-m-\alpha )-(n-m)^{\alpha +1})\bigg \{ \lambda ^{\alpha }N_m\left( 1 - \frac{N_m}{K^{\alpha }}\right) - \mu ^{\alpha } N_mP_m - \beta ^{\alpha }_1N_mI_m - \zeta ^{\alpha }_1 N_mM_m \bigg \} \bigg ], \\ I_{n+1}&=I_0+\frac{h^{\alpha }}{\Gamma (\alpha +2)} \sum \limits _{m=0}^n \bigg [ ((n-m+1)^{\alpha +1}-(n-m)^{\alpha }(n-m+\alpha +1))\bigg \{ \phi ^{\alpha }_1I_0 + \phi ^{\alpha }_2{N^2}_{m+1}^p - \phi ^{\alpha }_3I_{m+1}^p\\&\quad - \beta ^{\alpha }_2I_{m+1}^p P_{m+1}^p + \zeta ^{\alpha }_2 I_{m+1}^p M_{m+1}^p - \zeta ^{\alpha }_3 I_{m+1}^p R_{m+1}^p\bigg \}-((n-m+1)^{\alpha }(n-m-\alpha )-(n-m)^{\alpha +1})\bigg \{ \phi ^{\alpha }_1I_0 \\&\quad + \phi ^{\alpha }_2N_m^2 - \phi ^{\alpha }_3I_m - \beta ^{\alpha }_2I_m P_m + \zeta ^{\alpha }_2 I_m M_m - \zeta ^{\alpha }_3 I_m R_m \bigg \} \bigg ], \\ P_{n+1}&=P_0+\frac{h^{\alpha }}{\Gamma (\alpha +2)} \sum \limits _{m=0}^n \bigg [ ((n-m+1)^{\alpha +1}-(n-m)^{\alpha }(n-m+\alpha +1))\bigg \{ \gamma ^{\alpha } N_{m+1}^p P_{m+1}^p - \delta ^{\alpha } P_{m+1}^p\\&\quad - \beta ^{\alpha }_3I_{m+1}^p P_{m+1}^p + \zeta ^{\alpha }_4 P_{m+1}^p M_{m+1}^p \bigg \}-((n-m+1)^{\alpha }(n-m-\alpha )-(n-m)^{\alpha +1})\bigg \{ \gamma ^{\alpha } N_m P_m\\&\quad - \delta ^{\alpha } P(t) - \beta ^{\alpha }_3I_m P_m + \zeta ^{\alpha }_4 P_m M_m \bigg \} \bigg ], \\ M_{n+1}&=M_0+\frac{h^{\alpha }}{\Gamma (\alpha +2)} \sum \limits _{m=0}^n \bigg [ ((n-m+1)^{\alpha +1}-(n-m)^{\alpha }(n-m+\alpha +1))\bigg \{ \zeta ^{\alpha }_5 N_{m+1}^p M_{m+1}^p - \zeta ^{\alpha }_6 I_{m+1}^p M_{m+1}^p\\&\quad - \zeta ^{\alpha }_7 P_{m+1}^p M_{m+1}^p\ \bigg \}-((n-m+1)^{\alpha }(n-m-\alpha )-(n-m)^{\alpha +1})\bigg \{ \zeta ^{\alpha }_5 N_m M_m - \zeta ^{\alpha }_6 I_m M_m - \zeta ^{\alpha }_7 P_m M_m\ \bigg \} \bigg ],\\ R_{n+1}&=R_0+\frac{h^{\alpha }}{\Gamma (\alpha +2)} \sum \limits _{m=0}^n \bigg [ ((n-m+1)^{\alpha +1}-(n-m)^{\alpha }(n-m+\alpha +1))\bigg \{ \zeta ^{\alpha }_8 I_{m+1}^p R_{m+1}^p \bigg \}\\&\quad -((n-m+1)^{\alpha }(n-m-\alpha )-(n-m)^{\alpha +1})\bigg \{ \zeta ^{\alpha }_8 I_m R_m \bigg \} \bigg ]. \end{aligned}$$

Optimal system (14) can be solved by above algorithm. Similarly, we can use the following method to solve system (15):

$$\begin{aligned} \varvec{\lambda }_{n+1}&=\ \frac{h^{\alpha }}{\Gamma (\alpha +2)} \sum \limits _{m=0}^{n} \bigg \{ [(n-m+1)^{\alpha +1}-(n-m)^{\alpha }(n-m+\alpha +1)]\, \varvec{\Lambda }(t_{m+1}, {\textbf{y}}_{m+1}, \varvec{\lambda }_{m+1}^p) \\&\quad -[(n-m+1)^{\alpha }(n-m-\alpha )-(n-m)^{\alpha +1}]\, \varvec{\Lambda }(t_{m}, {\textbf{y}}_{m}, \varvec{\lambda }_{m})\bigg \}, \\ \varvec{\lambda }_{n+1}^p&=\frac{h^{\alpha }}{\Gamma (\alpha +1)} \sum \limits _{m=0}^{n} B_{\frac{n(n+1)}{2}+m+1}\,\varvec{\Lambda }(t_{m}, {\textbf{y}}_{m}, \varvec{\lambda }_{m}), \quad n=0, 1, \ldots , N, \end{aligned}$$

where $\varvec{\Lambda }(t, {\textbf{y}}(t), \varvec{\lambda }(t))$ is the vector of equations in the right side of system (15). After solving systems (1), (14), and (15), values of control variables can be updated by (16).

For numerical simulation, the following values have been considered for parameters and initial conditions in system (1):

$$\begin{aligned} \lambda&=0.3, \,\, \mu =0.01, \,\, \beta _1=0.01, \,\, \zeta _1=0.9, \,\, \phi _1=0.03, \,\, \phi _2=0.004, \,\, \phi _3=0.1, \,\, \beta _2=0.01, \,\, \zeta _2=0.458, \,\, K=10000, \,\,\\ \zeta _3&=0.8, \,\, \gamma =0.003, \,\, \delta =0.03, \,\, \beta _3=0.4, \,\, \zeta _4=0.045, \,\, \zeta _5=0.5, \,\, \zeta _6=0.6, \,\, \zeta _7=0.5, \,\, \zeta _8=0.6, \\ N_0&=1.5, \,\, I_0=0.7, \,\, P_0=0.3, \,\, M_0=0.1, \,\, R_0=0.1. \end{aligned}$$

**Figure 3:**
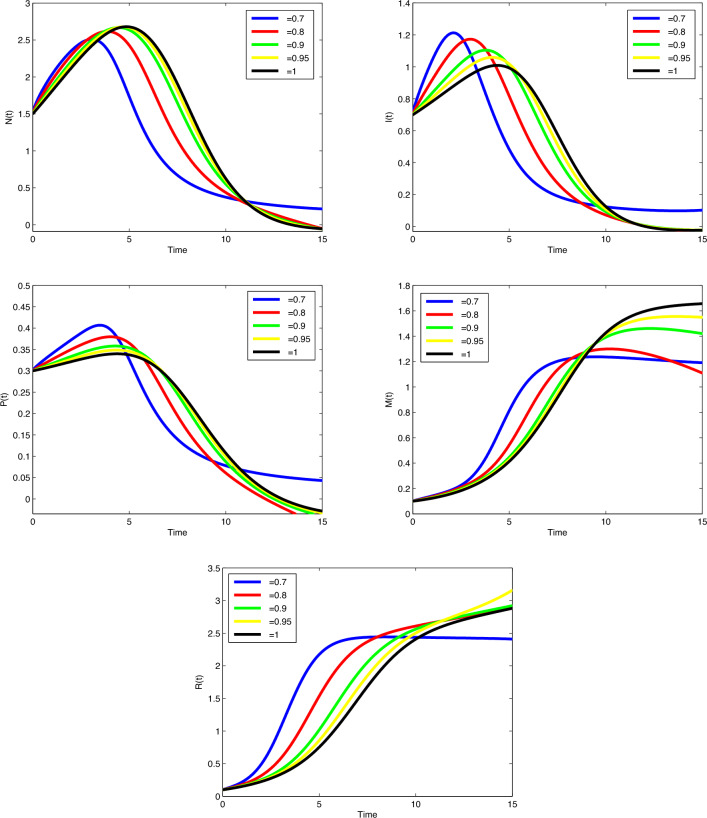
Plots of cancer cells, immune cells, spread cancer cells, genetic mutations, and enhanced immune cells in system (1) for different values of $\alpha$ and $N_0=1.5, I_0=0.7, P_0=0.3, M_0=0.1, R_0=0.1$.

**Figure 4:**
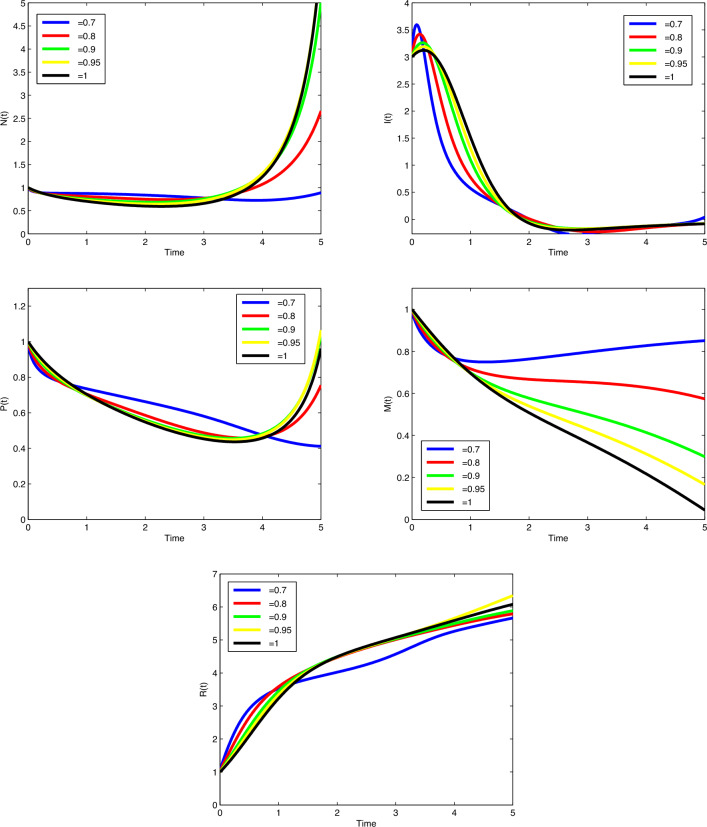
Plots of cancer cells, immune cells, spread cancer cells, genetic mutations, and enhanced immune cells in system (1) for different values of $\alpha$ and $N_0=1, I_0=3, P_0=1, M_0=1, R_0=1$.

**Figure 5:**
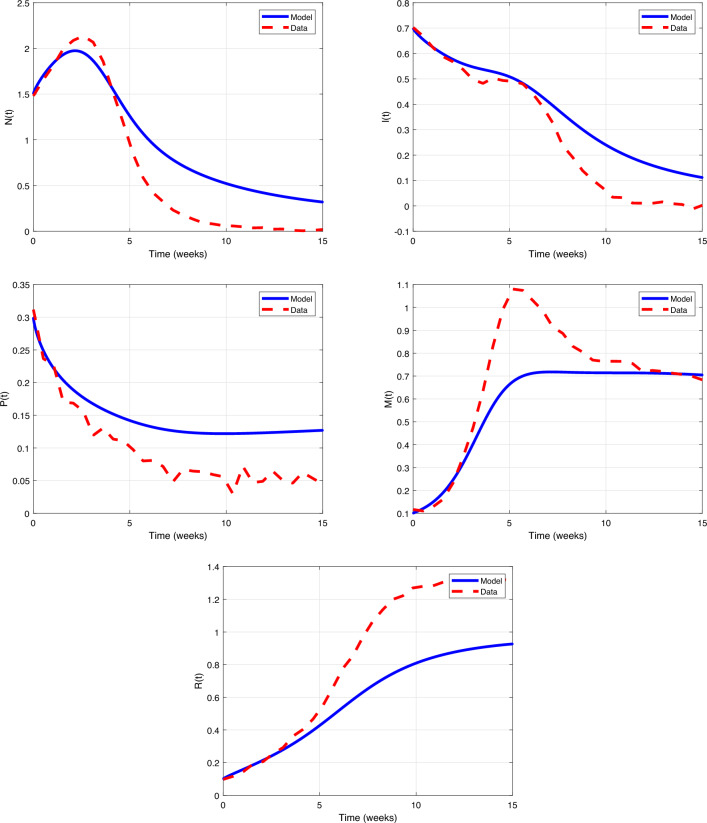
Validation comparison plot of model (1) versus synthetic data.

**Figure 6:**
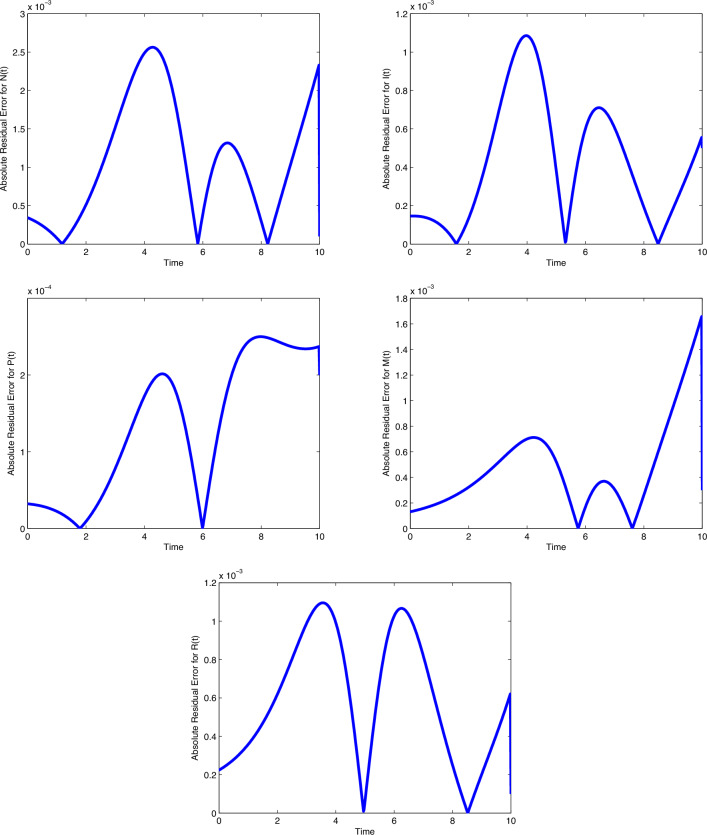
Plots of absolute residual errors for functions *N*(*t*), *I*(*t*), *P*(*t*), *M*(*t*),  and *R*(*t*) in system (1) for $\alpha =1$ and $N_0=1.5, I_0=0.7, P_0=0.3, M_0=0.1, R_0=0.1$.

**Figure 7:**
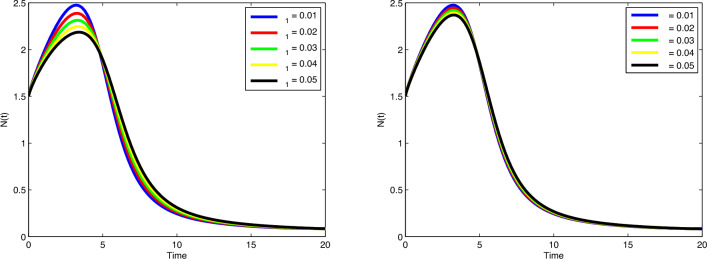
Plots of *N*(*t*) in system (1) for (Left) $\beta _1=0.01, 0.02, 0.03, 0.04, 0.05$, (Right) $\mu =0.01, 0.02, 0.03, 0.04, 0.05$, and $N_0=1.5, I_0=0.7, P_0=0.3, M_0=0.1, R_0=0.1$.

**Figure 8:**
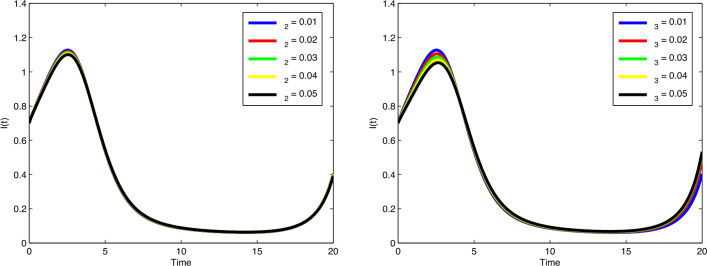
Plots of *I*(*t*) in system (1) for (Left) $\beta _2=0.01, 0.02, 0.03, 0.04, 0.05$, (Right) $\phi _3=0.01, 0.02, 0.03, 0.04, 0.05$, and $N_0=1.5, I_0=0.7, P_0=0.3, M_0=0.1, R_0=0.1$.

**Figure 9:**
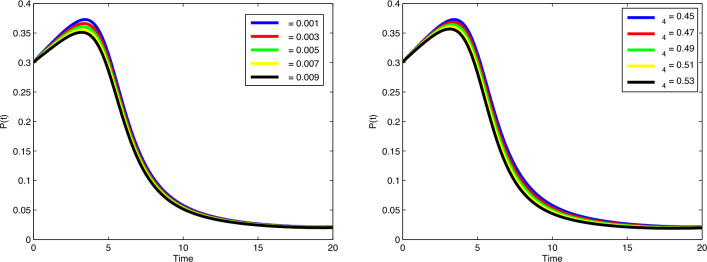
Plots of *P*(*t*) in system (1) for (Left) $\delta =0.001, 0.003, 0.005, 0.007, 0.009$, (Right) $\zeta _4=0.45, 0.47, 0.49, 0.51, 0.53$, and $N_0=1.5, I_0=0.7, P_0=0.3, M_0=0.1, R_0=0.1$.

**Figure 10:**
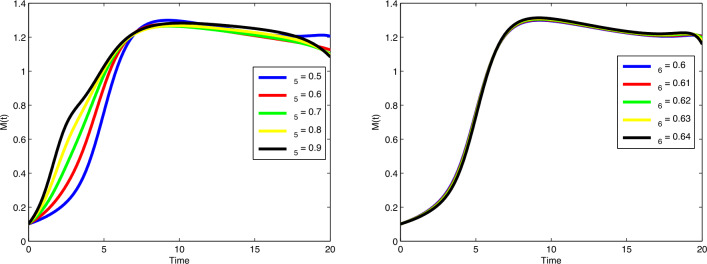
Plots of *M*(*t*) in system (1) for (Left) $\zeta _5=0.5, 0.6, 0.7, 0.8, 0.9$, (Right) $\zeta _6=0.6, 0.61, 0.62, 0.63, 0.64$, and $N_0=1.5, I_0=0.7, P_0=0.3, M_0=0.1, R_0=0.1$.

**Figure 11:**
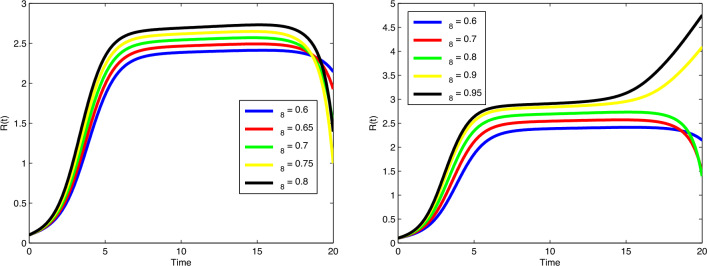
Plots of *R*(*t*) in system (1) for (Left) $\zeta _8=0.6, 0.65, 0.7, 0.75, 0.8$, (Right) $\zeta _8=0.6, 0.7, 0.8, 0.9, 0.95$, and $N_0=1.5, I_0=0.7, P_0=0.3, M_0=0.1, R_0=0.1$.

**Figure 12:**
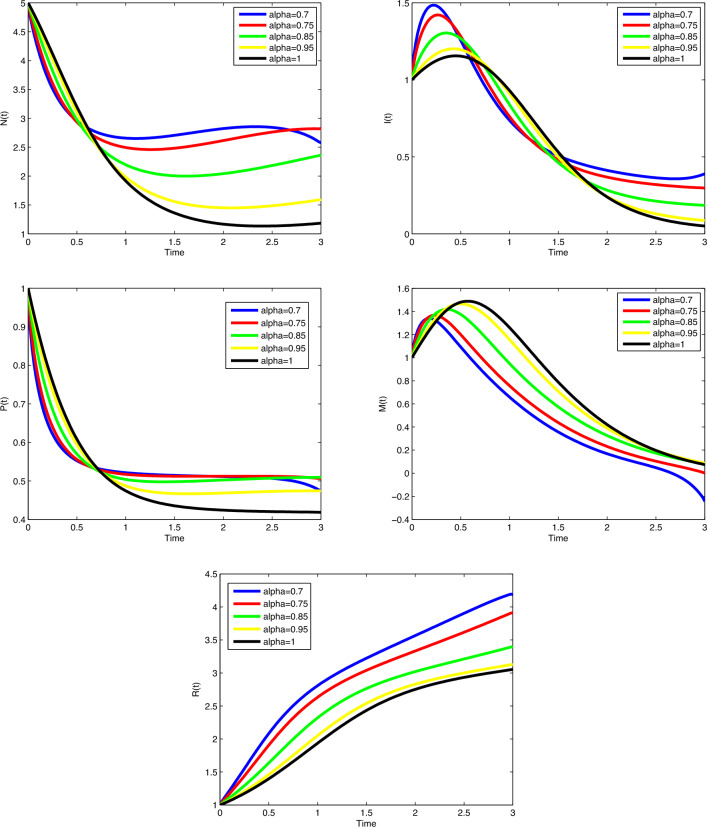
Plots of cancer cells, immune cells, spread cancer cells, genetic mutations, and enhanced immune cells in optimal system (14)–(17) for different values of $\alpha$ and $N_0=5, I_0=1, P_0=1, M_0=1, R_0=1$.

**Figure 13:**
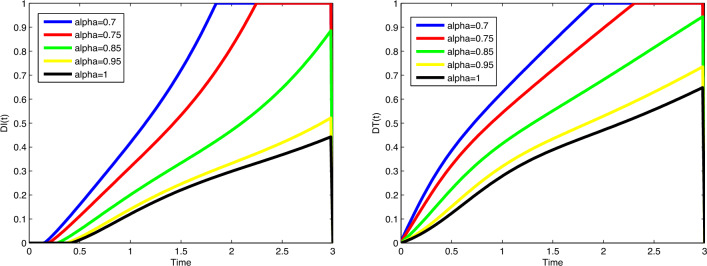
Plots of $D_I(t)$ and $D_T(t)$ in optimal system (14)–(17) for different values of $\alpha$ and $N_0=5, I_0=1, P_0=1, M_0=1, R_0=1$.

Figure 3 depicts the behaviour of the state variables in system (1) for $\alpha =0.7, 0.8, 0.9, 0.95, 1$. Figure 4 depicts the behaviour of the state variables in system (1) for $\alpha =0.7, 0.8, 0.9, 0.95, 1$, $N_0=1, I_0=3, P_0=1, M_0=1, R_0=1$. The number of cancer cells, spread cancer cells, and enhanced immune cells (in millions) increases and the number of immune cells starts to decrease after increasing. In all cases, the figures plotted for diverse values of $\alpha$ approach the figure plotted for $\alpha =1$. In Fig. 5, actual data points are compared to predicted values obtained from the suggested algorithms and the model. A coincidence between real data and numerical values is seen over the interval [0, 5] and on [5, 15], the simulated figures have an increasing or decreasing behaviour similar to the figures of the real data. Figures of state variables in optimal system (14)-(17) are seen in Fig. 12 for $\alpha =0.7, 0.8, 0.9, 0.95, 1$, $N_0=1, I_0=3, P_0=1, M_0=1, R_0=1$. In order to survey the validity of the numerical results obtained from the suggested model, the absolute residual errors for the state variables in System (1) are calculated. For this purpose, all terms in the equations of System (1) are shifted to the left side and obtained numerical values are substituted into them:

$$\begin{aligned} {\mathcal {R}}_1(t)&={_0^c}D_{t}^{\alpha }N(t) -\lambda ^{\alpha }N(t)\left( 1 - \frac{N(t)}{K^{\alpha }}\right) + \mu ^{\alpha } N(t)P(t) + \beta ^{\alpha }_1N(t)I(t) + \zeta ^{\alpha }_1 N(t)M(t) \approx 0,\\ {\mathcal {R}}_2(t)&={_0^c}D_{t}^{\alpha }I(t)- \phi ^{\alpha }_1I_0 - \phi ^{\alpha }_2N(t)^2 + \phi ^{\alpha }_3I(t) + \beta ^{\alpha }_2I(t)P(t) - \zeta ^{\alpha }_2 I(t)M(t) + \zeta ^{\alpha }_3 I(t)R(t)\approx 0,\\ {\mathcal {R}}_3(t)&={_0^c}D_{t}^{\alpha }P(t) -\gamma ^{\alpha } N(t)P(t) + \delta ^{\alpha } P(t) + \beta ^{\alpha }_3I(t)P(t) - \zeta ^{\alpha }_4 P(t)M(t)\approx 0,\\ {\mathcal {R}}_4(t)&= {_0^c}D_{t}^{\alpha }M(t)+\zeta ^{\alpha }_5 N(t)M(t) + \zeta ^{\alpha }_6 I(t)M(t) + \zeta ^{\alpha }_7 P(t)M(t)\approx 0,\\ {\mathcal {R}}_5(t)&={_0^c}D_{t}^{\alpha }R(t)- \zeta ^{\alpha }_8 I(t)R(t)\approx 0, \end{aligned}$$

where ${\mathcal {R}}_i(t), i=1, 2, 3, 4, 5$ are residual functions. Thus, we have the following system for $\alpha =1$:

$$\begin{aligned} {\mathcal {R}}_1(t_j)&=\frac{N_{j+1}-N_j}{h} -\lambda ^{\alpha }N_j\left( 1 - \frac{N_j}{K^{\alpha }}\right) + \mu ^{\alpha } N_j P_j + \beta ^{\alpha }_1N_j I_j + \zeta ^{\alpha }_1 N_j M_j \approx 0,\\ {\mathcal {R}}_2(t_j)&=\frac{I_{j+1}-I_j}{h}- \phi ^{\alpha }_1I_0 - \phi ^{\alpha }_2N_j^2 + \phi ^{\alpha }_3I_j + \beta ^{\alpha }_2I_jP_j - \zeta ^{\alpha }_2 I_j M_j + \zeta ^{\alpha }_3 I_j R_j\approx 0,\\ {\mathcal {R}}_3(t_j)&=\frac{P_{j+1}-P_j}{h} -\gamma ^{\alpha } N_j P_j + \delta ^{\alpha } P_j + \beta ^{\alpha }_3I_j P_j - \zeta ^{\alpha }_4 P_j M_j \approx 0,\\ {\mathcal {R}}_4(t_j)&= \frac{M_{j+1}-M_j}{h}+\zeta ^{\alpha }_5 N_j M_j + \zeta ^{\alpha }_6 I_j M_j + \zeta ^{\alpha }_7 P_j M_j \approx 0,\\ {\mathcal {R}}_5(t_j)&=\frac{R_{j+1}-R_j}{h}- \zeta ^{\alpha }_8 I_j R_j\approx 0, \end{aligned}$$

for $j=0, 1, \cdots , N$. Figures of absolute residual errors are depicted in Fig. 6. As expected, the values of the residual functions are small. In other words, the obtained numerical results from the suggested algorithms are getting close to the exact data if they are available. As another criterion to measure the validity of the proposed model, the sensitivity of the model to some of its parameters is investigated. Hence, the plots of state variables can be seen in Figs. 7, 8, 9, 10 and 11 for $\alpha =0.8$, initial conditions $N_0=1.5, I_0=0.7, P_0=0.3, M_0=0.1, R_0=0.1$, and different values of diverse parameters. As can be seen, by increasing the values of parameters, figures of state functions are not divergent. In Figure 7 by increasing values of $\beta _1$ and $\mu$, the number of cancer cells are decreasing gradually. In Fig. 8, by increasing the value of $\beta _2$, the number of immune cells sounds constant, while by increasing the value of $\phi _3$, the number of immune cells decreases gradually. Cancer cells spread in a similar way by increasing values of $\delta$ and $\zeta _4$ in Fig. 9. In Fig. 10, no variation observes in the behaviour of *M*(*t*) by increasing the value of $\zeta _6$. In Fig. 11, with the increase of the value of $\zeta _8$, the number of enhanced cytotoxic immune cells increases. The figures of the control variables $D_I(t)$ and $D_T(t)$ are depicted in Fig. 13. The number of cancers and the spread of cancer cells is increasing with time (weeks).

Similarly, to estimate values of $D_T(t), D_I(t), u_T(t)$, and $u_I(t)$ in system (18), we consider the following Hamiltonian function:

30 $$\begin{aligned} {\textsf{H}}(t)=&a_1^{\alpha } N(t)+a_2^{\alpha } I(t)+a_3^{\alpha } P(t)+ a_4^{\alpha } M(t)+a_5^{\alpha } R(t)+b_1^{\alpha } D_I^2(t) +b_2^{\alpha } D_T^2(t) +c_1^{\alpha } u_I^2(t)+c_2^{\alpha } u_T^2(t)\\&+\Lambda _1(t) \bigg \{ \lambda ^{\alpha }N(t)\left( 1 - \frac{N(t)}{K^{\alpha }}\right) - \mu ^{\alpha } N(t)P(t) - \beta ^{\alpha }_1N(t)I(t) - \zeta ^{\alpha }_1 N(t)M(t) - \chi ^{\alpha }_1 N(t)\left( D_I(t) + u_I(t)\right) \bigg \} \\&+\Lambda _2(t) \bigg \{ \phi ^{\alpha }_1I_0 + \phi ^{\alpha }_2N^2(t) - \phi ^{\alpha }_3I(t) - \beta ^{\alpha }_2I(t)P(t) + \zeta ^{\alpha }_2 I(t)M(t) - \zeta ^{\alpha }_3 I(t)R(t) + \chi ^{\alpha }_2 I(t)\left( D_T(t) + u_T(t)\right) \bigg \} \\&+\Lambda _3(t) \bigg \{ \gamma ^{\alpha } N(t)P(t) - \delta ^{\alpha } P(t) - \beta ^{\alpha }_3I(t)P(t) + \zeta ^{\alpha }_4 P(t)M(t) - \chi ^{\alpha }_3 P(t)\left( D_T(t) + u_T(t)\right) \bigg \}\\&+\Lambda _4(t) \bigg \{ \zeta ^{\alpha }_5 N(t)M(t) - \zeta ^{\alpha }_6 I(t)M(t) - \zeta ^{\alpha }_7 P(t)M(t) + \chi ^{\alpha }_4 M(t)(D_T(t) + u_T(t)) + \chi ^{\alpha }_5 M(t)\left( D_I(t) + u_I(t)\right) \bigg \}\\&+ \Lambda _5(t) \bigg \{\zeta ^{\alpha }_8 I(t)R(t) + \chi ^{\alpha }_6 R(t)\left( D_I(t) + u_I(t)\right) \bigg \}, \end{aligned}$$

where $\Lambda _i(t), i=1, \ldots , 5$ are adjoint variables. If $D^*_T, D^*_I, u^*_T,$ and $u^*_I$ are optimal values of control variables, then the optimal system, utilizing Hamiltonian (30), will be as follows:

31 $$\begin{aligned} _{0}^{c}D_{t}^{\alpha }N(t)&= \lambda ^{\alpha }N(t)\left( 1 - \frac{N(t)}{K^{\alpha }}\right) - \mu ^{\alpha } N(t)P(t) - \beta ^{\alpha }_1N(t)I(t) - \zeta ^{\alpha }_1 N(t)M(t) - \chi ^{\alpha }_1 N(t)\left( D^*_I(t) + u^*_I(t)\right) , \nonumber \\ _{0}^{c}D_{t}^{\alpha }I(t)&= \phi ^{\alpha }_1I_0 + \phi ^{\alpha }_2N^2(t) - \phi ^{\alpha }_3I(t) - \beta ^{\alpha }_2I(t)P(t) + \zeta ^{\alpha }_2 I(t)M(t) - \zeta ^{\alpha }_3 I(t)R(t) + \chi ^{\alpha }_2 I(t)\left( D^*_T(t) + u^*_T(t)\right) , \nonumber \\ _{0}^{c}D_{t}^{\alpha }P(t)&= \gamma ^{\alpha } N(t)P(t) - \delta ^{\alpha } P(t) - \beta ^{\alpha }_3I(t)P(t) + \zeta ^{\alpha }_4 P(t)M(t) - \chi ^{\alpha }_3 P(t)\left( D^*_T(t) + u^*_T(t)\right) , \nonumber \\ _{0}^{c}D_{t}^{\alpha }M(t)&= \zeta ^{\alpha }_5 N(t)M(t) - \zeta ^{\alpha }_6 I(t)M(t) - \zeta ^{\alpha }_7 P(t)M(t) + \chi ^{\alpha }_4 M(t)(D^*_T(t) + u^*_T(t)) + \chi ^{\alpha }_5 M(t)\left( D^*_I(t) + u^*_I(t)\right) , \nonumber \\ _{0}^{c}D_{t}^{\alpha }R(t)&= \zeta ^{\alpha }_8 I(t)R(t) + \chi ^{\alpha }_6 R(t)\left( D^*_I(t) + u^*_I(t)\right) , \end{aligned}$$

32 $$\begin{aligned} \frac{d \Lambda _1(t)}{dt}&=-a_1^{\alpha }-\Lambda _1(t)\bigg [\lambda ^{\alpha } -\frac{2 \lambda ^{\alpha }}{K^{\alpha }}N(t)-\mu ^{\alpha }P(t)-\beta _1^{\alpha } I(t)-\zeta _1^{\alpha } M(t)-\chi _1^{\alpha } (D_I(t)+u_I(t)\bigg ]\nonumber \\&\quad -2 \phi _2^{\alpha } \Lambda _2(t) N(t)-\gamma ^{\alpha } \Lambda _3(t) P(t)-\zeta _5^{\alpha } \Lambda _4(t) M(t), \nonumber \\ \frac{d \Lambda _2(t)}{dt}&=-a_2^{\alpha }+\beta _1^{\alpha } \Lambda _1(t)N(t)-\Lambda _2(t)\bigg [ -\phi _3^{\alpha }-\beta _2^{\alpha } P(t)+\zeta _2^{\alpha } M(t)-\zeta _3^{\alpha } R(t)+\chi _2^{\alpha } (D_T(t)+u_T(t)) \bigg ]\nonumber \\&\quad + \beta _3^{\alpha } \Lambda _3(t) P(t) +\zeta _6^{\alpha } \Lambda _4(t) M(t) -\zeta _8^{\alpha } \Lambda _5(t) R(t),\nonumber \\ \frac{d \Lambda _3(t)}{dt}&=-a_3^{\alpha }+\mu ^{\alpha } \Lambda _1(t) N(t) +\beta _2 ^{\alpha } \Lambda _2(t) I(t)-\Lambda _3(t) \bigg [ \gamma ^{\alpha } N(t)-\delta ^{\alpha } -\beta _3^{\alpha } I(t)+\zeta _4^{\alpha } M(t)-\chi _3^{\alpha } (D_T(t)\nonumber \\&\quad +u_T(t)) \bigg ]+\zeta _7^{\alpha } \Lambda _4(t) M(t), \nonumber \\ \frac{d \Lambda _4(t)}{dt}&=-a_4^{\alpha }+\zeta _1^{\alpha } \Lambda _1(t) N(t) -\zeta _2^{\alpha } \Lambda _3(t) P(t)-\Lambda _4(t)\bigg [ \zeta _5^{\alpha } N(t)-\zeta _6^{\alpha } I(t)-\zeta _7^{\alpha } P(t) +\chi _4^{\alpha } (D_T(t)\nonumber \\&\quad +u_T(t))+\chi _5^{\alpha } (D_I(t)+u_I(t))\bigg ],\nonumber \\ \frac{d \Lambda _5(t)}{dt}&=-a_5^{\alpha }+\zeta _3^{\alpha } \Lambda _2(t) I(t)-\Lambda _5(t) \bigg [ \zeta _8^{\alpha } I(t) +\chi _6^{\alpha } (D_I(t)+u_I(t))\bigg ], \end{aligned}$$

33 $$\begin{aligned} D_I^*&=\min \{\max \{0, \Delta _I\}, 1\}, \quad D_T^*=\min \{\max \{0, \Delta _T\}, 1\},\nonumber \\ u_I^*&=\min \{\max \{0, \Psi _I\}, 1\}, \quad u_T^*=\min \{\max \{0, \Psi _T\}, 1\}, \end{aligned}$$

where

$$\begin{aligned} \Delta _I&=\frac{\chi _1^{\alpha } \Lambda _1(t) N(t)-\chi _5^{\alpha } \Lambda _4(t) M(t) -\chi _6^{\alpha } \Lambda _5(t) R(t) }{2 b_1 ^{\alpha }},\\ \Delta _T&=-\frac{\chi _2^{\alpha } \Lambda _2(t) I(t)-\chi _3^{\alpha } \Lambda _3(t) P(t) +\chi _4^{\alpha } \Lambda _4(t) M(t) }{2 b_2 ^{\alpha }},\\ \Psi _I&=\frac{\chi _1^{\alpha } \Lambda _1(t) N(t)-\chi _5^{\alpha } \Lambda _4(t) M(t) -\chi _6^{\alpha } \Lambda _5(t) R(t) }{2 c_1 ^{\alpha }},\\ \Psi _T&=-\frac{\chi _2^{\alpha } \Lambda _2(t) I(t)-\chi _3^{\alpha } \Lambda _3(t) P(t) +\chi _4^{\alpha } \Lambda _4(t) M(t) }{2 c_2 ^{\alpha }}. \end{aligned}$$

**Figure 14:**
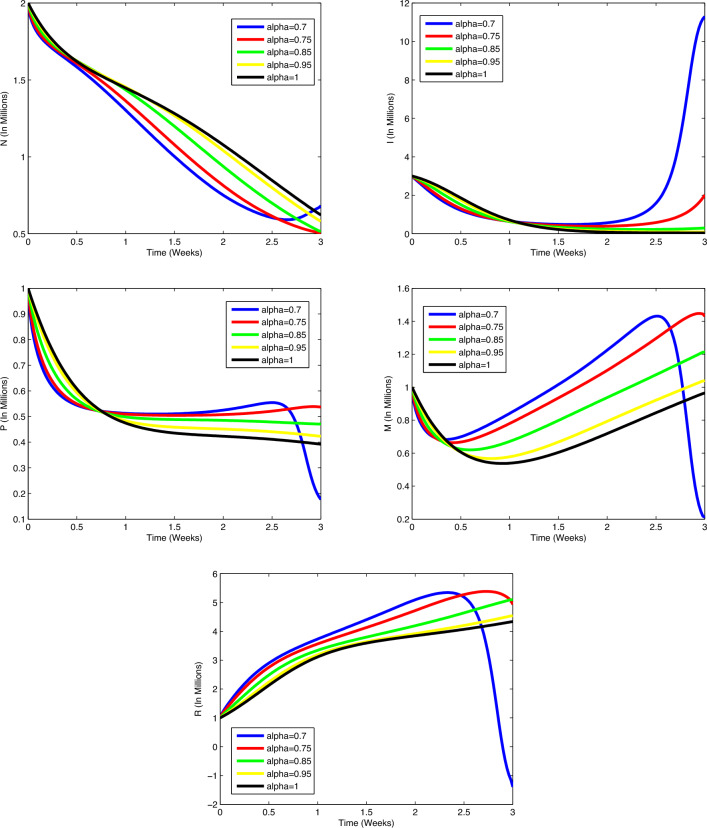
Plots of cancer cells, immune cells, spread cancer cells, genetic mutations, and enhanced immune cells in optimal system (31)–(33) for different values of $\alpha$ and $N_0=2, I_0=3, P_0=1, M_0=1, R_0=1$.

**Figure 15:**
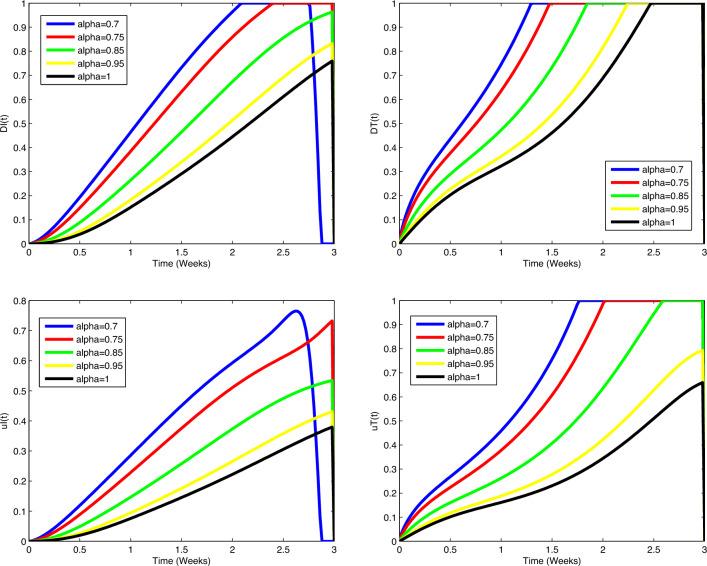
Plots of $D_I(t)$, $D_T(t)$, $u_I(t)$, and $u_T(t)$ in optimal system (31)–(33) for different values of $\alpha$ and $N_0=2, I_0=3, P_0=1, M_0=1, R_0=1$.

**Figure 16:**
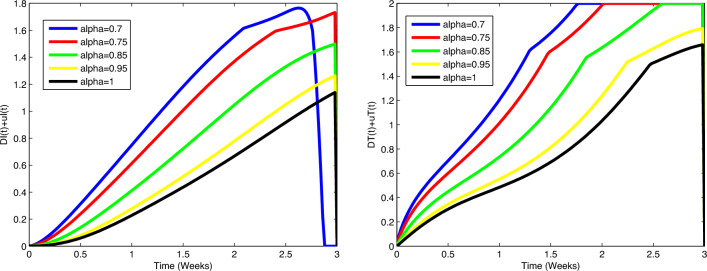
Plots of $D_I(t)+u_I(t)$ and $D_T(t)+u_T(t)$ in optimal system (31)–(33) for different values of $\alpha$.

After solving problem (31)–(33) using the proposed predictor-corrector method, figures of state and control variables are depicted in Figs. 14 and 15. The number of spread cancer cells remains almost constant after a decreasing trend. The behaviour of control signals ($u_I(t)$ and $u_T(t)$) after adjusting the drug dosages ($D_I(t)$ and $D_T(t)$) is seen in Fig. 16.

**Figure 17:**
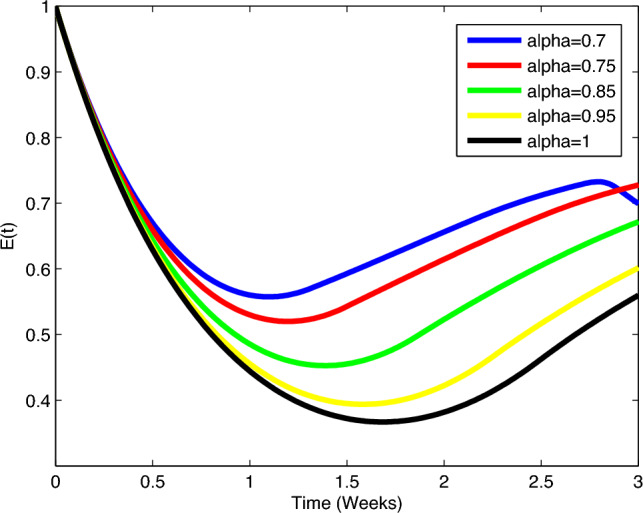
The long-term effects *E*(*t*) for different values of $\alpha$.

The model’s long-term effects *E*(*t*), defined by evolution equation (22), are seen in Fig. 17 for $\alpha =0.7, 0.75, 0.85, 0.95, 1$, $\theta _1=1, \theta _2=0.3, \theta _3=0.5$, and $E_0=1$. 

**Figure 18:**
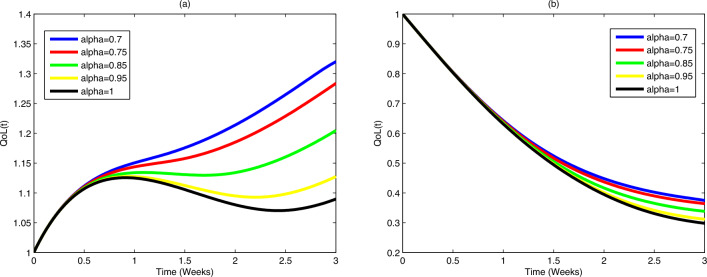
Survivorship considerations *QoL*(*t*) for $h=0.01, N_0=2, I_0=3, P_0=M_0=R_0=1$, and: (**a**) $\delta _0=0.6, \eta =0.25$, (**b**) $\delta _0=0.3, \eta =0.7$.

Figures of the quality of life *QoL*(*t*) introduced in (23) are seen in Fig. 18 for different values of parameters and $\alpha$ and $Q_0=1$.

Now, by having approximate values of $D_I, D_T$, And *E*, we can compute values of the direct costs ($C_{\text {direct}}$), indirect costs ($C_{\text {indirect}}$), and cost-benefit ratio (CBR) for $h=0.01$ and $T_f=3$ by the Trapezoidal method to evaluate the integral in (19) and (20). Values of these quantities are listed in Table 3 for different values of $\alpha$, $\delta _0=0.6$, and $\eta =0.25$.

**Table 3:** Direct costs, indirect costs, and cost-benefit ratio for different values of $\alpha$

$\alpha$	0.7	0.75	0.85	0.95	1
$C_{\text {direct}}$	45.80198089	49.59691439	52.20094648	51.80341784	51.01911228
$C_{\text {indirect}}$	3.52874962	3.47455262	3.37727573	3.29607094	3.26157215
${\text {CBR }}$	0.07153248	0.06546932	0.06076617	0.05982035	0.06008716

## Result and discussion

Analytical results have shown that system (1) is well-defined and has a unique solution. Figures 3 and 4 describe the endemic dynamics of the lung cancer model without treatment or control. The validation of the model as depicted in Fig. 5, demonstrates overall effectiveness in capturing the dynamics of the biological system under study. Moreso, the absolute residual error plots provide further insight into the performance of our model by showcasing the discrepancies between the model predictions and the actual data, as shown in Fig. 6. The plots comparing the model predictions with the actual (synthetic) data show strong agreement with variables, indicating high accuracy, and this is further corroborated in the sensitivity plots of Figs. 7, 8, 9, 10 and 11. The number of cancer cells and cancer cells that spread to other parts of the body increases rapidly. The optimized treatment strategy as depicted through Figs. 12, 13 and 14 in the fractional-order lung cancer model demonstrates encouraging outcomes across multiple variables. Notably, the reduction in the number of cancer cells (*N*(*t*)) signifies the efficacy of the combined immunotherapy and targeted therapy in controlling primary tumor growth. This outcome aligns with the overarching goal of inhibiting cancer progression, highlighting the potential clinical impact of the optimization strategy. A particularly positive outcome is the observed decline in the number of cancer cells that have spread (*P*(*t*)) after optimization. This indicates that the treatment strategy not only targets the primary tumor but also exhibits efficacy in curtailing the metastatic potential of cancer cells. Limiting metastasis is a critical objective in cancer treatment, as it significantly influences patient prognosis and long-term survival. While the levels of immune cells (*I*(*t*)) display a staggered response after optimization, several factors may contribute to this observation. The intricate dynamics of the tumor microenvironment, characterized by immune evasion mechanisms employed by cancer cells, could influence the overall immune response. Further refinement of the treatment strategy may be necessary to enhance the recruitment and activation of immune cells, addressing the complexities of the immune-tumor interaction. Similarly, the stabilized levels of genetic mutations (*M*(*t*)) after optimization suggest that the treatment strategy effectively controls the emergence and propagation of mutated cancer cells. Genetic mutations often contribute to tumor aggressiveness, and their containment is a positive outcome for long-term therapeutic success. A particularly promising result is the increase in immune cells (*R*(*t*)) with enhanced cytotoxic activity after optimization. This signifies that the treatment strategy positively influences the immune response, potentially activating cytotoxic *T* cells that play a crucial role in targeting and eliminating cancer cells. The enhancement of immune cytotoxicity is a key aspect of fostering anti-tumor immunity. Biological intricacies, such as the tumor microenvironment and the dynamics of genetic mutations, warrant careful consideration for a comprehensive understanding of the observed results. Fine-tuning optimization parameters, including drug dosages and intervention strengths, may further optimize the treatment strategy. Additionally, experimental validation and comparison with clinical data would provide valuable insights, bridging the computational results with real-world implications. The results underscore the potential of the optimized treatment strategy in achieving key therapeutic goals, including primary tumor control, metastasis limitation, and augmentation of immune cytotoxicity. Further exploration and refinement of the model, guided by experimental evidence and clinical insights, will contribute to the development of robust and effective personalized cancer treatment approaches. The observed behavior in the drug dosages, as depicted in Fig. 15, where $D_I(t)$ reaches 1 more sharply than $D_T(t)$, followed by a rapid decline in both to eventually reach 0, holds important implications for the optimized treatment strategy. This pattern indicates a targeted and focused application of immunotherapy (represented by $D_I(t)$) that rapidly achieves its intended impact, while the targeted therapy (represented by $D_T(t)$) follows suit with a slightly delayed and sustained effect. The rapid increase in immunotherapy dosage ($D_I(t)$) suggests an immediate and intensified effort to enhance the immune response against cancer cells. Immunotherapy is designed to stimulate the patient’s immune system, particularly cytotoxic *T* cells, to recognize and attack cancer cells more effectively. The abrupt rise in $D_I(t)$ reflects a swift initiation of this immune-boosting intervention. In contrast, the targeted therapy dosage ($D_T(t)$) exhibits a more gradual rise. Targeted therapies often involve drugs designed to interfere with specific molecular targets involved in cancer growth and spread. The slower ascent of $D_T(t)$ may signify a careful and sustained application, allowing for a more controlled inhibition of cancer cell pathways targeted by the therapy. The subsequent sharp decline of both $D_I(t)$ and $D_T(t)$ to ultimately reach 0 implies a temporally limited and controlled treatment regimen. This observed trend aligns with the concept of optimizing drug dosages to maximize therapeutic impact while minimizing potential side effects and long-term toxicities associated with prolonged drug exposure. Immunotherapy Primacy: The prompt escalation and subsequent rapid decline in immunotherapy suggest that its primary role might be in initiating a potent immune response against cancer cells. This aligns with the strategy of harnessing the body’s natural defenses to target and eliminate cancer. The more sustained elevation of targeted therapy dosage implies an ongoing effort to interfere with specific cancer cell pathways. This sustained application may be essential for suppressing the molecular mechanisms that drive cancer progression and survival. The coordinated rise and fall of both drug dosages indicate a temporally optimized treatment approach. The goal is to achieve an optimal therapeutic effect during a defined timeframe while minimizing the risk of resistance development or adverse effects associated with prolonged drug exposure. The observed dynamics in drug dosages reflect a nuanced and temporally optimized treatment strategy. The distinct profiles of $D_I(t)$ and $D_T(t)$ suggest a deliberate sequencing of interventions, leveraging the strengths of immunotherapy for rapid immune activation and targeted therapy for sustained molecular interference. This temporally optimized approach may contribute to enhanced treatment efficacy and reduced long-term toxicities, aligning with the principles of precision medicine in cancer therapy. The observed behavior in the control signals as depicted in Fig. 16, where $u_I(t)$ approaches but does not reach 1 before sharply reducing to 0, while $u_T(t)$ reaches 1 and then sharply declines to 0 for various values of alpha, provides valuable insights into the dynamics of the control actions in the optimized treatment strategy. The fact that $u_I(t)$ approaches but does not reach 1 indicates a careful and controlled manipulation of the immunotherapy dosage ($D_I(t)$) through the PID controller. The value not reaching 1 suggests a nuanced adjustment, possibly to avoid excessive or abrupt changes in immunotherapy dosage, thereby maintaining a balance between treatment efficacy and potential side effects. In contrast, the behavior of $u_T(t)$, where it reaches 1 before sharply declining to 0, suggests a more decisive and pronounced manipulation of the targeted therapy dosage ($D_T(t)$). The reaching of 1 implies a significant amplification of the targeted therapy, possibly to maximize its inhibitory effects on specific cancer cell pathways. The subsequent sharp decline indicates a controlled withdrawal of this intervention. The controlled approach of $u_I(t)$ aligns with the principle of careful modulation of immunotherapy. While immunotherapy is a powerful tool in stimulating the immune system, its excessive activation may lead to undesirable side effects. The observed modulation indicates a sophisticated control mechanism, optimizing the immune response without inducing unnecessary risks. The reaching and subsequent sharp decline of $u_T(t)$ suggest a decisive and temporally limited application of targeted therapy. This strategy may be aimed at achieving a potent inhibition of cancer cell-specific pathways, followed by a prompt withdrawal to mitigate potential long-term toxicities associated with prolonged exposure to targeted agents. The PID controller’s role is evident in these dynamics, showcasing its ability to finely tune and balance the control signals based on the error signals (differences between desired and actual states). The controller’s actions contribute to the optimization of drug dosages over time, considering both the rapid but controlled nature of immunotherapy and the more decisive application of targeted therapy. The observed behaviors of $u_I(t)$ and $u_T(t)$ highlight the PID controller’s role in orchestrating a nuanced and temporally optimized treatment strategy. The controlled approach of $u_I(t)$ and the decisive nature of $u_T(t)$ underscore the importance of balancing treatment efficacy with safety considerations. These dynamics contribute to the overall precision and adaptability of the proposed treatment approach, aligning with the principles of personalized and optimized cancer therapy. The observed behavior where $D_I(t) + u_I(t)$ reaches 1.8 and $D_T(t) + u_T(t)$ reaches 2 in the optimal system (Equations (31)–(33)) for different values of $\alpha$, as shown in Fig. 16 provides insights into the dynamic adjustments of drug dosages under the optimized treatment strategy. The value exceeding 1 for $D_I(t) + u_I(t)$ implies an augmentation beyond the baseline immunotherapy dosage. This amplification could be a strategic response to boost the immune response against cancer cells. The excess beyond 1 suggests an intentional overshooting, potentially leveraging the body’s ability to handle a temporary surge in immunotherapeutic effects. Similarly, the value reaching 2 for $D_T(t) + u_T(t)$ signifies a pronounced escalation of the targeted therapy dosage beyond its baseline. This substantial increase may be aimed at achieving an intensified inhibition of specific cancer cell pathways targeted by the therapy. The value of 2 indicates a deliberate and significant amplification of the targeted treatment. Immunotherapy Intensification: The overshooting observed in $D_I(t) + u_I(t)$ suggests a strategic intensification of immunotherapy, possibly to induce a robust and rapid immune response against cancer cells. This strategy aligns with the understanding that immunotherapy’s effectiveness may benefit from intermittent periods of heightened activation. The reaching of 2 in $D_T(t) + u_T(t)$ indicates a purposeful and intensified application of targeted therapy. This heightened dosage could be designed to maximize the inhibitory effects on specific molecular pathways associated with cancer progression. The strategy might involve a brief but potent exposure to achieve a therapeutic impact. The dynamics of exceeding 1 and 2 in $D_I(t) + u_I(t)$ and $D_T(t) + u_T(t)$, respectively, and subsequently returning to 0 reflect the dynamic and adaptive nature of the treatment strategy. The system seems to undergo strategic escalations followed by controlled de-escalations, contributing to the overall adaptability of the treatment approach. The PID controller plays a pivotal role in orchestrating these dynamic changes. The overshooting and subsequent decline are indicative of the PID controller’s ability to respond to error signals, providing a mechanism for finely tuning and optimizing drug dosages in real-time. The observed behaviors of $D_I(t) + u_I(t)$ and $D_T(t) + u_T(t)$ underscore the dynamic and strategic nature of the optimized treatment approach. The intentional overshooting and subsequent controlled decline reveal the sophistication of the PID-controlled system in achieving a balance between treatment efficacy and potential side effects. These dynamics contribute to the precision and adaptability of the proposed treatment strategy in the context of personalized and optimized cancer therapy. Table 3 presents direct costs ($C_{\text {direct}}$), indirect costs ($C_{\text {indirect}}$), and the cost-benefit ratio (*CBR*) for various values of $\alpha$ in the context of the fractional-order lung cancer model, and illustrated in Figs. 17 and 18. Each of these metrics contributes to the economic evaluation and efficiency assessment of the proposed treatment strategy. Direct costs encompass expenses directly associated with the implementation of the treatment strategy, including drug costs and medical services. The values of $C_{\text {direct}}$ increase with higher values of $\alpha$, indicating that as the fractional order of the derivatives in the system dynamics increases, there is a corresponding escalation in the direct costs of the treatment. This could be attributed to the complexity and precision required in the implementation of the treatment strategy for higher-order fractional systems. Indirect costs capture factors influencing societal well-being and are often associated with the quality of life. In contrast to direct costs, $C_{\text {indirect}}$ exhibits a decreasing trend as $\alpha$ increases. This suggests that, for higher fractional orders, the societal burden and associated indirect costs may decrease, possibly indicating a more effective and targeted treatment approach. The cost-benefit ratio provides a comprehensive measure of the economic efficiency of the treatment strategy. It is the ratio of indirect costs to the sum of direct and indirect costs. The *CBR* values follow a declining trend as $\alpha$ increases. This implies that, despite the increase in direct costs, the overall benefits, as measured by improvements in quality of life, outpace the escalating costs. A decreasing *CBR* indicates an economically favorable scenario where the benefits derived from the treatment strategy surpass the combined direct and indirect costs. The variations in direct costs, indirect costs, and the cost-benefit ratio highlight the sensitivity of the treatment strategy to the fractional order of the system. Understanding these variations is crucial for optimizing resource allocation and achieving cost-effective treatment outcomes. The decreasing trend in *CBR* with increasing $\alpha$ suggests a careful balance between the economic costs of treatment and the therapeutic benefits. It indicates that, as the system becomes more intricate (higher fractional order), the treatment strategy remains economically viable, emphasizing its adaptability and efficiency. The cost-benefit analysis provides decision-makers with valuable insights into the economic implications of the proposed treatment strategy. It helps guide the allocation of resources and facilitates informed choices in the selection of treatment parameters. This result contribute to the understanding of the treatment strategy’s cost dynamics, balancing direct and indirect costs while emphasizing the importance of considering the fractional order in optimizing both therapeutic and economic outcomes. The memory effect in the cancer cell dynamics is observed through the fractional-order derivative terms in the equation for *N*(*t*). These terms incorporate the historical concentrations and interactions of cancer cells, influencing the current growth or decline. The memory effect captures the persistence of the impact of past tumor sizes and interactions on the current state of cancer cells. Biologically, this reflects the tumor’s ability to “remember” its growth history, influencing the trajectory of cancer cell dynamics. Fractional-order terms in the equations governing immune cells *I*(*t*)) introduce memory, reflecting the historical response of the immune system to cancer cells and treatments. The memory effect in immune cell dynamics acknowledges the lasting influence of past immune responses on the current state. This aligns with the biological concept that the immune system retains information about previous encounters, shaping its ongoing behavior. The fractional-order terms in the equations describing genetic mutations *M*(*t*)) imply that past mutations contribute to the current state of the system. Memory in genetic mutations highlights the cumulative impact of past genetic alterations on the evolution of cancer cells. Biologically, this mirrors the concept that genetic changes accumulate over time, influencing the genetic landscape of the tumor. The integral terms in the PID controller equations introduce memory, considering the accumulation of past errors in drug dosages. The integral terms represent the memory effect in adapting drug dosages. Biological Interpretation: The memory effect in the PID controller aligns with the clinical reality that treatment decisions are influenced by past responses. Clinicians, akin to the PID controller, adjust drug dosages based on historical errors to improve future therapeutic outcomes. The fractional-order terms in the differential equations governing long-term effects *E*(*t*)) and quality of life *QoL*(*t*)) reflect memory in the persistence of treatment-related impacts and survivorship outcomes. Memory in long-term effects and survivorship is biologically relevant as it considers the enduring consequences of past treatments on the patient’s well-being. The system “remembers” past exposures, contributing to a holistic understanding of extended treatment outcomes. The identified memory effects reinforce the biological relevance of the fractional-order lung cancer model, capturing the persistent influence of past events on the current and future behavior of the system. Clinically, understanding memory effects can guide treatment decisions, emphasizing the importance of past responses in shaping future interventions. The memory effects identified in the results enhance the model’s realism and align with biological and clinical principles. They provide a comprehensive representation of the system’s dynamics by considering the enduring impact of past states on the evolving behavior of the fractional-order lung cancer model.

## Conclusion

In the pursuit of enhancing cancer treatment strategies, the proposed fractional-order model for optimizing combination therapy in heterogeneous lung cancer stands as a promising framework. This study specifically integrates immunotherapy and targeted therapy to minimize side effects, presenting a holistic approach to achieving therapeutic efficacy while mitigating potential drawbacks. This study introduces a fractional-order model for optimizing combination therapy in heterogeneous lung cancer, integrating immunotherapy and targeted therapy to minimize side effects and enhance therapeutic efficacy. The model’s analytical validation confirms its reliability in capturing cancer progression and treatment dynamics. The optimized combination therapy significantly reduces cancer and metastatic cell populations, demonstrating the potential of integrating immunotherapy and targeted therapy. The treatment strategy, guided by a PID controller, dynamically adjusts drug dosages to minimize side effects while enhancing the immune response. This balanced approach ensures a carefully modulated application of therapies, aiming to harness their strengths without prolonged adverse effects. Economic analysis reveals a favorable cost-benefit ratio, suggesting that despite higher direct costs associated with increased fractional orders, the overall therapeutic benefits outweigh the costs. This finding supports the model’s practicality and efficiency in a real-world clinical context. Additionally, the identification of memory effects in cancer and immune cell dynamics, as well as in drug dosages, adds a layer of realism to the model. These memory effects reflect the enduring impact of past treatments on current and future behavior, aligning with clinical observations. The research highlights the potential of this model to tailor cancer treatments by incorporating real-time data and individual patient characteristics, thereby enhancing personalized therapy. Future work should focus on clinical trials to validate these findings and refine the model parameters for practical implementation. The goal is to translate these computational insights into tangible benefits for cancer patients, advancing personalized cancer treatment strategies. This interdisciplinary approach bridges the gap between computational modeling and clinical applications, promising significant advancements in the field of personalized medicine. The novelty of this research lies in its innovative use of fractional-order differential equations to capture the complexities and memory effects inherent in cancer systems, which are often overlooked in traditional models. By integrating immunotherapy and targeted therapy with a sophisticated PID control strategy, this study offers a unique, adaptive approach to cancer treatment that is both personalized and dynamically responsive to patient-specific conditions. The inclusion of economic considerations and real-time data further enhances the model’s applicability and potential impact on clinical practice. Future promising research should focus on conducting clinical trials to further validate the model’s predictions and refine its parameters for real-world application. Additionally, a detailed comparative analysis with existing models, while beyond the scope of this study, is recommended for future studies to better contextualize our findings within the broader landscape of cancer treatment research. Exploring the integration of advanced control strategies, such as machine learning algorithms, could enhance the adaptability and precision of treatment. Expanding the model to incorporate various cancer types and incorporating emerging biomarkers and patient-specific data will pave the way for even more personalized and effective therapeutic strategies. This interdisciplinary research bridges computational modeling and clinical application, promising significant advancements in personalized cancer treatment.

## Data Availability

All pertinent data utilized in this investigation are referenced within the manuscript and are available upon request from the corresponding author (CI).

## References

[1] Tao, M.H. Epidemiology of lung cancer. Lung Cancer Imaging 4–1 (2019).

[2] Schabath, M. B. & Cote, M. L. Cancer progress and priorities: Lung cancer. *Cancer Epidemiol. Biomark. Prev.* **28**(10), 1563–1579 (2019). 10.1158/1055-9965.EPI-19-0221 PMC6777859 31575553

[3] Wahla, A. S., Zoumot, Z., Uzbeck, M., Mallat, J., Souilamas, R. & Shafiq, I. The Journey for Lung Cancer Screening where we Stand Today. Open Respir. Med. J. 16, (2022). 10.2174/18743064-v16-e2207060 PMC10156027 37273952

[4] Are, C. *et al.* A review of global cancer burden: Trends, challenges, strategies, and a role for surgeons. *J. Surg. Oncol.* **107**(2), 221–226 (2013). 22926725 10.1002/jso.23248

[5] Gomez, D. R. & Liao, Z. Non-small cell lung cancer (NSCLC) and small cell lung cancer (SCLC). In *Target Volume Delineation and Field Setup: A Practical Guide for Conformal and Intensity-Modulated Radiation Therapy* 87–103 (Springer, 2012).

[6] Bradley, J. D. *et al.* Gross tumor volume, critical prognostic factor in patients treated with three-dimensional conformal radiation therapy for non-small-cell lung carcinoma. *Int. J. Radiat. Oncol. Biol. Phys.* **52**(1), 49–57 (2002). 11777621 10.1016/S0360-3016(01)01772-2

[7] Cheung, W. K. & Nguyen, D. X. Lineage factors and differentiation states in lung cancer progression. *Oncogene* **34**(47), 5771–5780 (2015). 25823023 10.1038/onc.2015.85 PMC7597437

[8] Masuda, A. & Takahashi, T. Chromosome instability in human lung cancers: Possible underlying mechanisms and potential consequences in the pathogenesis. *Oncogene* **21**(45), 6884–6897 (2002). 12362271 10.1038/sj.onc.1205566

[9] Chen, Z., Fillmore, C. M., Hammerman, P. S., Kim, C. F. & Wong, K. K. Non-small-cell lung cancers: A heterogeneous set of diseases. *Nat. Rev. Cancer* **14**(8), 535–546 (2014). 25056707 10.1038/nrc3775 PMC5712844

[10] Sun, S., Schiller, J. H. & Gazdar, A. F. Lung cancer in never smokers-a different disease. *Nat. Rev. Cancer* **7**(10), 778–790 (2007). 17882278 10.1038/nrc2190

[11] Herbst, R. S., Heymach, J. V. & Lippman, S. M. Lung cancer. *N. Engl. J. Med.* **359**(13), 1367 (2008). 18815398 10.1056/NEJMra0802714 PMC10662965

[12] Lemjabbar-Alaoui, H., Hassan, O. U., Yang, Y. W. & Buchanan, P. Lung cancer: Biology and treatment options. *Biochimica et Biophysica Acta (BBA)-Rev. Cancer* **1856**(2), 189–210 (2015). 10.1016/j.bbcan.2015.08.002 PMC4663145 26297204

[13] Spiro, S. G. & Porter, J. C. Lung cancer-where are we today? Current advances in staging and nonsurgical treatment. *Am. J. Respir. Crit. Care Med.* **166**(9), 1166–1196 (2002). 12403687 10.1164/rccm.200202-070SO

[14] Jones, C. M., Brunelli, A., Callister, M. E. & Franks, K. N. Multimodality treatment of advanced non-small cell lung cancer: Where are we with the evidence?. *Curr. Surg. Rep.* **6**, 1–11 (2018). 10.1007/s40137-018-0202-0 PMC5805813 29456881

[15] Akiyama, Y. *et al.* Advantages and disadvantages of combined chemotherapy with carmustine wafer and bevacizumab in patients with newly diagnosed glioblastoma: A single-institutional experience. *World Neurosurg.* **113**, e508–e514 (2018). 29476996 10.1016/j.wneu.2018.02.070

[16] König, J. *et al.* Radiotherapy effects on early breast cancer survival in observational and randomized studies: A systematic analysis of advantages, disadvantages and differences between the two study types. *Breast Cancer* **23**, 415–424 (2016). 25585654 10.1007/s12282-014-0579-2

[17] Mortezaee, K. *et al.* Synergic effects of nanoparticles-mediated hyperthermia in radiotherapy/chemotherapy of cancer. *Life Sci.* **269**, 119020 (2021). 33450258 10.1016/j.lfs.2021.119020

[18] Tiwari, P. *et al.* Surface modification strategies in translocating nano-vesicles across different barriers and the role of bio-vesicles in improving anticancer therapy. *J. Control. Release* **363**, 290–348 (2023). 37714434 10.1016/j.jconrel.2023.09.016

[19] Singh, K., Bhori, M., Kasu, Y. A., Bhat, G. & Marar, T. Antioxidants as precision weapons in war against cancer chemotherapy induced toxicity-Exploring the armoury of obscurity. *Saudi Pharm. J.* **26**(2), 177–190 (2018). 30166914 10.1016/j.jsps.2017.12.013 PMC6111235

[20] Prasanna, P. G. *et al.* Normal tissue protection for improving radiotherapy: Where are the Gaps?. *Transl. Cancer Res.* **1**(1), 35 (2012). 22866245 PMC3411185

[21] Xuan, L., Bai, C., Ju, Z., Luo, J., Guan, H., Zhou, P. K. & Huang, R. Radiation-targeted immunotherapy: A new perspective in cancer radiotherapy. Cytokine Growth Factor Rev. (2023). 10.1016/j.cytogfr.2023.11.003 38061920

[22] Ladoire, S., Rébé, C. & Ghiringhelli, F. Associating immunotherapy and targeted therapies: Facts and hopes. *Clin. Cancer Res.* **29**(7), 1183–1193 (2023). 36445399 10.1158/1078-0432.CCR-22-1184

[23] Shah, M. A. *et al.* Immunotherapy and targeted therapy for advanced gastroesophageal cancer: ASCO guideline. *J. Clin. Oncol.* **41**(7), 1470–1491 (2023). 36603169 10.1200/JCO.22.02331

[24] Liu, K., Zhu, Y. & Zhu, H. Immunotherapy or targeted therapy as the first-line strategies for unresectable hepatocellular carcinoma: A network meta-analysis and cost-effectiveness analysis. *Front. Immunol.* **13**, 1103055 (2023). 36713376 10.3389/fimmu.2022.1103055 PMC9874298

[25] Abaza, A., Idris, F.S., Shaikh, H. A., Vahora, I., Moparthi, K. P., Al Rushaidi, M.T., Muddam, M. Programmed cell death protein 1 (PD-1) and programmed cell death ligand 1 (PD-L1) immunotherapy: A promising breakthrough in cancer therapeutics. Cureus 15(9), (2023). 10.7759/cureus.44582 PMC10475160 37667784

[26] Thorat, V. M., Surale-Patil, S. A., Singh, L., Chavda, A. V. & Salve, P. S. Immunotherapy revolution in oncology current status and future directions. *J. ReAttach Ther. Dev. Divers.* **6**(1), 737–742 (2023).

[27] Araghi, M. *et al.* Recent advances in non-small cell lung cancer targeted therapy; an update review. *Cancer Cell Int.* **23**(1), 162 (2023). 37568193 10.1186/s12935-023-02990-y PMC10416536

[28] Wang, R. C. & Wang, Z. Precision medicine: Disease subtyping and tailored treatment. *Cancers* **15**(15), 3837 (2023). 37568653 10.3390/cancers15153837 PMC10417651

[29] Ye, F. *et al.* Advancements in clinical aspects of targeted therapy and immunotherapy in breast cancer. *Mol. Cancer* **22**(1), 105 (2023). 37415164 10.1186/s12943-023-01805-y PMC10324146

[30] Vanneman, M. & Dranoff, G. Combining immunotherapy and targeted therapies in cancer treatment. *Nat. Rev. Cancer* **12**(4), 23251 (2012). 10.1038/nrc3237 PMC3967236 22437869

[31] Agarwal, P., Baleanu, D., Chen, Y., Momani, S. & Machado, J. T. Fractional calculus, In ICFDA, International Workshop on Advanced Theory and Applications of Fractional Calculus. Amman (2019).

[32] Muresan, C. I., Birs, I. R., Dulf, E. H., Copot, D. & Miclea, L. A review of recent advances in fractional-order sensing and filtering techniques. *Sensors* **21**(17), 5920 (2021). 34502811 10.3390/s21175920 PMC8434365

[33] Gokbulut, N., Amilo, D. & Kaymakamzade, B. Fractional SVIR model for COVID-19 under Caputo derivative. *J. Biometry Stud.* **1**(2), 58–64 (2021). 10.29329/JofBS.2021.349.04

[34] Amilo, D., Sadri, K., Kaymakamzade, B. & Hincal, E. A mathematical model with fractional-order dynamics for the combined treatment of metastatic colorectal cancer. *Commun. Nonlinear Sci. Numer. Simulat.* **130**, 107756 (2023). 10.1016/j.cnsns.2023.107756

[35] Shah, K. & Abdeljawad, T. On complex fractal-fractional order mathematical modeling of CO 2 emanations from energy sector. *Phys. Scr.* **99**(1), 015226 (2023). 10.1088/1402-4896/ad1286

[36] Sinan, M. *et al.* Analysis of the mathematical model of cutaneous leishmaniasis disease. *Alex. Eng. J.* **72**, 117–134 (2023). 10.1016/j.aej.2023.03.065

[37] Khan, Z. A., Shah, K., Abdalla, B. & Abdeljawad, T. A numerical study of complex dynamics of a chemostat model under fractal-fractional derivative. *Fractals* **31**(08), 2340181 (2023). 10.1142/S0218348X23401813

[38] Ahmed, S., Shah, K., Jahan, S. & Abdeljawad, T. An efficient method for the fractional electric circuits based on Fibonacci wavelet. *Results Phys.* **52**, 106753 (2023). 10.1016/j.rinp.2023.106753

[39] Alinei-Poiana, T., Dulf, E. H. & Kovacs, L. Fractional calculus in mathematical oncology. *Sci. Rep.* **13**(1), 10083 (2023). 37344605 10.1038/s41598-023-37196-9 PMC10284913

[40] Özköse, F. *et al.* A fractional modeling of tumor-immune system interaction related to Lung cancer with real data. *Eur. Phys. J. Plus* **137**, 1–28 (2022).34909366 10.1140/epjp/s13360-021-02254-6

[41] Hassani, H. *et al.* A study on fractional tumor-immune interaction model related to lung cancer via generalized Laguerre polynomials. *BMC Med. Res. Methodol.* **23**(1), 189 (2023). 37605131 10.1186/s12874-023-02006-3 PMC10440950

[42] Amilo, D., Kaymakamzade, B. & Hincal, E. A fractional-order mathematical model for lung cancer incorporating integrated therapeutic approaches. *Sci. Rep.* **13**(1), 12426 (2023). 37528101 10.1038/s41598-023-38814-2 PMC10394091

[43] Nath, B. J., Sadri, K., Sarmah, H. K. & Hosseini, K. An optimal combination of antiretroviral treatment and immunotherapy for controlling HIV infection. *Math. Comput. Simul.* **217**(2024), 226–243 (2024). 10.1016/j.matcom.2023.10.012

[44] Burden, R. L., Faires, J. D. & Burden, A. M. *Numerical Analysis* 10th edn. (Cengage Learning, 2015).

[45] He, S., Wang, H. & Sun, K. Solutions and memory effect of fractional-order chaotic system: A review. *Chin. Phys. B* **31**(6), 060501 (2022). 10.1088/1674-1056/ac43ae

[46] Xie, W., Wu, W. Z., Liu, C. & Goh, M. Generalized fractional grey system models: The memory effects perspective. *ISA Trans.* **126**, 36–46 (2022). 34366121 10.1016/j.isatra.2021.07.037

[47] Chevalier, M., Gómez-Schiavon, M., Ng, A. H. & El-Samad, H. Design and analysis of a proportional-integral-derivative controller with biological molecules. *Cell Syst.* **9**(4), 338–353 (2019). 31563473 10.1016/j.cels.2019.08.010

[48] Yu, C. C. Features of Proportional-Integral-Derivative Control, Autotuning of PID Controllers: A Relay Feedback Approach, 9–21 (2006).

[49] Bergholz, J. S., Wang, Q., Kabraji, S. & Zhao, J. J. Integrating immunotherapy and targeted therapy in cancer treatment: Mechanistic insights and clinical implications. *Clin. Cancer Res.* **26**(21), 5557–5566 (2020). 32576627 10.1158/1078-0432.CCR-19-2300 PMC7641965

[50] Tong, X., Dong, C. & Liang, S. Mucin1 as a potential molecule for cancer immunotherapy and targeted therapy. *J. Cancer* **15**(1), 54 (2024). 38164273 10.7150/jca.88261 PMC10751670

[51] Ascierto, P. A. *et al.* Sequential immunotherapy and targeted therapy for metastatic BRAF V600 mutated melanoma: 4-year survival and biomarkers evaluation from the phase II SECOMBIT trial. *Nat. Commun.* **15**(1), 146 (2024). 38167503 10.1038/s41467-023-44475-6 PMC10761671

[52] Sontakke, B. R. & Shaikh, A. S. Properties of Caputo operator and its applications to linear fractional differential equations. *Int. J. Eng. Pic. Appl.* **5**(5), 22–27 (2015).

[53] Asjad, M. I. Novel fractional differential operator and its application in fluid dynamics. *J. Prime Res. Math.* **16**(2), 67–79 (2020).

[54] Özköse, F. *et al.* A fractional modeling of tumor-immune system interaction related to lung cancer with real data. *Eur. Phys. J. Plus* **137**, 40 (2022). 10.1140/epjp/s13360-021-02254-6

